# Single-cell lipidomics with high structural specificity by mass spectrometry

**DOI:** 10.1038/s41467-021-23161-5

**Published:** 2021-05-17

**Authors:** Zishuai Li, Simin Cheng, Qiaohong Lin, Wenbo Cao, Jing Yang, Minmin Zhang, Aijun Shen, Wenpeng Zhang, Yu Xia, Xiaoxiao Ma, Zheng Ouyang

**Affiliations:** 1 State Key Laboratory of Precision Measurement Technology and Instruments, Department of Precision Instrument, Tsinghua University, Beijing, China; 2 MOE Key Laboratory of Bioorganic Phosphorus Chemistry & Chemical Biology, Department of Chemistry, Tsinghua University, Beijing, China; 3 Division of Anti-tumor Pharmacology, State Key Laboratory of Drug Research, Shanghai Institute of Materia Medica, Chinese Academy of Sciences, Shanghai, China; 4 Department of Chemistry, Purdue University, West Lafayette, IN USA

**Keywords:** Lipidomics, Mass spectrometry, Bioanalytical chemistry

## Abstract

Single-cell analysis is critical to revealing cell-to-cell heterogeneity that would otherwise be lost in ensemble analysis. Detailed lipidome characterization for single cells is still far from mature, especially when considering the highly complex structural diversity of lipids and the limited sample amounts available from a single cell. We report the development of a general strategy enabling single-cell lipidomic analysis with high structural specificity. Cell fixation is applied to retain lipids in the cell during batch treatments prior to single-cell analysis. In addition to tandem mass spectrometry analysis revealing the class and fatty acyl-chain for lipids, batch photochemical derivatization and single-cell droplet treatment are performed to identify the C=C locations and *sn*-positions of lipids, respectively. Electro-migration combined with droplet-assisted electrospray ionization enables single-cell mass spectrometry analysis with easy operation but high efficiency in sample usage. Four subtypes of human breast cancer cells are correctly classified through quantitative analysis of lipid C=C location or *sn*-position isomers in ~160 cells. Most importantly, the single-cell deep lipidomics strategy successfully discriminates gefitinib-resistant cells from a population of wild-type human lung cancer cells (HCC827), highlighting its unique capability to promote precision medicine.

## Abstract (web summary)

Analyzing the lipidomes of single cells remains a challenge. Here, the authors present a strategy to identify class, fatty acyl-chain, C=C locations and *sn*-positions of lipids in single cells, and use their method to identify individual gefitinib-resistant cells in a wild-type lung cancer cell population.

## Introduction

Single-cell analysis is essential for the study of many fundamental biological phenomena, revealing the cellular heterogeneity during cell development. Multifarious techniques have been developed for applying various omics for single cell analysis, including genomics, proteomics, and metabolomics^1–10^. Among those omics, the proteome and metabolome respond to external stimuli more rapidly and usually are harder to be detected or identified due to their complex structures and low biomarker abundances in a single cell^11^. Mass spectrometry (MS) has become a method of choice for single-cell metabolome analysis in consideration of its high sensitivity and high structural identification capabilities^12–20^.

However, comprehensive structural characterization and quantitation of a relatively large number of metabolites, for example, lipids, is still difficult at the single-cell level. The chemical structures of lipids are very complex and a significant amount of efforts have been put in the discovery of interesting chemistry (e.g., photochemical reactions and epoxidation) for elucidating lipid structures using MS^21^. Paternò–Büchi (PB) reaction combined with tandem MS is one of the most effective protocols for the detailed characterization and relative quantitation of lipids at carbon–carbon double bond (C=C) location and *sn*-position levels^22–26^. Epoxidation is also common in deep lipidomic analysis with C=C location specificity, owing to its universal accessibility^27–31^. The composition of C=C location isomers or *sn*-position isomers from human plasma, tissues and cell populations have been obtained using these chemical derivatizations^32–37^. In the past few years, some MS analysis methods have been developed to profile lipids in single cells, such as inkjet printed microarrays^38^, probe ESI (electrospray ionization)^39^, single-probe MS^40^, T probe^41^, secondary ion MS^42^, matrix-assisted laser desorption/ionization (MALDI) MS^43^, and cyESI-MS^44^. The requirement of special media solutions and devices, however, hindered the coupling of chemical derivatization with these methods. MS image of lipid C=C location isomers by PB-MALDI-MS/MS has been reported at the resolution of single-cell size^45,46^, but this image could not point to the cell accurately due to the differences between regular image pixel and irregular cell morphology. Recently, MS method coupled with PB reaction were reported to identify C=C locations of lipids in single cell^47^, but relative quantitation of isomers has never been done and its unique biological significance was not demonstrated.

**Fig. 1:**
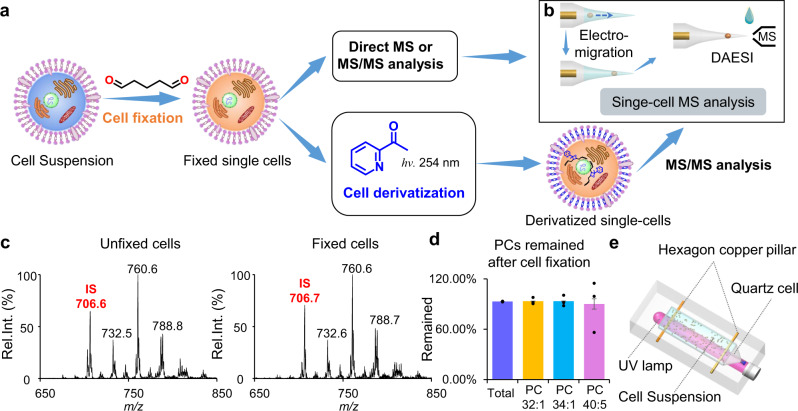
Workflow of single-cell lipidomics with high structural specificity. **a** Workflow of single-cell fixation and optional derivatization for subsequent MS analysis. **b** Workflow of single-cell MS analysis method that allowed multi-round sampling and data acquisition. DAESI: droplet-assisted electrospray ionization. **c** Precursor ion scan of *m/z* 184 spectrum of the extract of unfixed (left) and fixed (right) MDA-MB-231 cells. Internal standard (IS): PC 15:0-15:0 (*m/z* 706). **d** Remain of PCs after fixation. Error bar represents the standard deviation, *n* = 3. **e** Experimental setup of collective single cells photochemical derivatization. Source data are provided in a Source Data file.

In this work, we develop a protocol with shotgun MS analysis for single-cell lipidomics with high structural specificity including C=C locations or *sn-*positions (Fig. 1a). The cells are first fixed by glutaraldehyde to prevent cell lysis in salt-free solution. Batch photochemical derivatization (PB reaction with hydro-soluble 2-acetylpyridine) are performed to allow a comprehensive lipid characterization with C=C location specificity through later single-cell MS/MS analysis. The *sn*-position of phosphatidylcholines (PCs) are obtained through MS/MS analysis of bicarbonate adduct ions. The relative quantitation of the major lipid C=C location and *sn*-position isomers reveals the degree of heterogeneity differences among four human breast cancer cell lines, including MDA-MB-231, MDA-MB-468, MCF-7, and BT-474. The distinct capability of the developed method is that only the relative quantitation of lipid C=C location isomers can discriminate gefitinib-resistant single cells in wild-type non-small-cell lung cancer (NSCLC) cell population, demonstrating its unique significance for precision medicine.

## Results

### Cell fixation, cell manipulation, and collective derivatization

An efficient method for cell manipulation and subsequent MS analysis based on electro-migration and electroporation was previously developed for analyzing cells with cell walls, including fungus, algae, and protozoa^48^. However, to extend this method for mammalian cells, isotonic buffer (e.g., phosphate-buffered saline, PBS) needs to be used to prevent the lysis of the cell membrane, which is incompatible with electrospray ionization. To circumvent this problem and confine lipids within the cell through the batch cell manipulation and derivatization prior to MS analysis, cell fixation was proposed as the first step of the analysis procedure. During fixation, amines in cellular proteins react with glutaraldehyde to form cross-linked proteins in cytoskeleton, which effectively prevents the cell lysis in water^49^. By using precursor ion scan of *m/z* 184, it was demonstrated that the recovery of PCs after fixation was more than 90% (Fig. 1c, d and Supplementary Fig. 1a–d). The recovery ratio was not influenced by the concentration of the cells (Supplementary Fig. 1e, f). It should be noted that lipids containing amine groups, like PEs, would be lost, due to their reactions with glutaraldehyde (Supplementary Fig. 1g, h). This fixation made it possible to manipulate a single mammalian cell in salt-free solvents such as pure water, which is more friendly to ESI-MS analysis in comparison with PBS.

For lipid structure identification, chemical derivatization such as PB reaction was particularly useful; however, this would cause damages to the cell membrane, leading to cell lysis and cross-contaminations among single cells during lipid derivatization process. This problem could now be solved with the cell fixation, which also provided a relatively high local concentration of lipids for PB reaction. In previous studies for lipid analysis involving PB reactions, acetone was one of the most popular derivatizing reagents for the identification and relative quantitation of unsaturated lipids^34^. However, acetone was usually used at relatively high percentages, which would lead to the breakdown of cell membrane. Ideally, to avoid lipid dissolution and diffusion, lipid derivatization should be carried out in an aqueous environment. We therefore chose a serials of hydro-soluble carbonyl compounds as PB reagents, including 2-acetylpyridine (2-AP), 3-AP, and 4-AP. Cells were derivatized in a quartz cell under 254 nm UV irradiation (Fig. 1d). Among all APs, 2-AP has the highest conversion yield (Supplementary Fig. 2a) and the corresponding PB product ion offers the highest relative intensities of the diagnostic ions as well (Supplementary Fig. 2b–d). Therefore, 2-AP was finally chosen for lipid derivatization in single cells, with an optimal reaction time of 8 min and an optimal concentration of 100 mM (Supplementary Fig. 2e, f). After derivatization, cells were washed twice using pure water to remove excess 2-AP and would be individually analyzed subsequently.

### Single-cell MS analysis

**Fig. 2:**
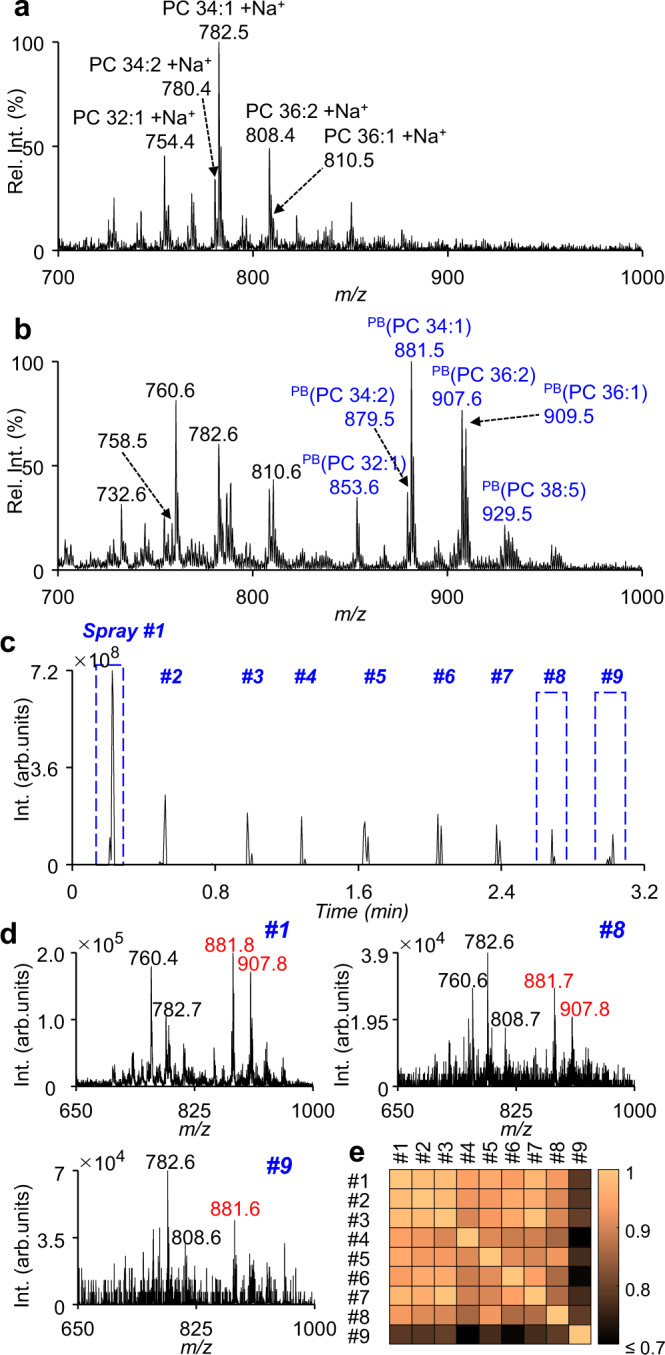
MS spectrum of a single MDA-MB-231 cell. **a**, **b** MS spectra of lipids from a single MDA-MB-231cell without (**a**) and with (**b**) photochemical derivatization (the *m/z* values in blue: PB products of lipids). **c** Total ion chromatogram (TIC) of a single MDA-MB-231 cell sampled 9 times by on-demand DAESI-MS after cell fixation and photochemical derivatization. **d** Average MS spectra of the 1st, 8th, and 9th samplings (the *m/z* values in red: PB products of lipids). **e** Pairwise correlation coefficients calculated using the intensities of PB product ions of each spray. Source data are provided in a Source Data file.

Previously, we have developed method for manipulating and analyzing single cells using electro-migration^48^. Using a modified method, individual cell could be sent to a capillary tip and multi-round sampling of the cell could be performed for MS or MS/MS data acquisition. Following the fixation, the cells were washed with water and then diluted to a concentration of ~ 10^4^ cells/mL. For single-cell MS analysis, ~ 0.5 μL of cell suspension was transferred by pipette to a capillary with a pulled tip for nanoESI. To drive the cell to the front end of the capillary, a direct current (DC) voltage of −1.3 kV was applied by a stainless-steel electrode (Fig. 1b). To achieve a better sample consumption efficiency, following the solvent evaporation in the nanoESI tip, droplet-assisted electrospray ionization (DAESI) was used to enable single-cell MS analysis^50^. The limit of detection of DAESI was reported to be 50 attomole, which is sensitive enough for single-cell analysis^51^. A droplet (~50 μL) of assistant solvent (methanol:acetonitrile = 1:1 (v/v) with 1% formic acid) was applied to the front of the capillary to initiate DAESI of the single cell. Such a solvent condition has been demonstrated to be efficient for both lipid extraction and nanoESI^51^. The sample ionization could be readily synchronized with ion introduction for mass analysis so the sample consumption was reduced. A variety of PCs were detected with high abundances from single mammalian cells, e.g., the human breast cancer cells MDA-MB-231 (Fig. 2a). As for analysis of single cells after photochemical derivatization, the peaks of PB products of unsaturated lipids (marked as ^PB^M) were clearly observed, each with a *m/z* value increase of 121 Da (Fig. 2b). The ion intensity of PB products was even a little higher than that of the corresponding intact lipid, possibly due to the higher proton affinity of the pyridine in 2-AP. Changes in the relative intensity of sodium and proton adduct ions of PCs before and after PB reaction are possibly due to solution acidification after PB reaction and variation of sodium in cells.

It has been reported that DAESI can enable high MS sensitivity at reduced sample consumption^51^, lipids and their PB products from a single cell could be more effectively detected and structurally characterized. In addition, multiple rounds of sampling of an individual cell could be performed using DAESI, since the compounds were only consumed when the assisted droplet was applied, which was operated in an on-demand ionization mode. In the analysis of a derivatized single MDA-MB-231 cell, for instance, even upon the 8th sampling, the signal-to-noise ratios (S/N ratios) of PB products for PC 34:1 and PC 36:2 (at *m/z* 881.7 and 907.8) were still high enough for MS/MS analysis (Fig. 2c, d). The spectra of other spray could be seen in Supplementary Fig. 3a. Pairwise correlation coefficients calculated using the intensities of PB product ions of each spray were ≥0.85 among the first 8 sprays (Fig. 2e). This experiment was repeated 5 times and the results were similar (Supplementary Fig. 3b–i), indicating that at least 8 species of lipids of various *m/z* values could be readily analyzed from a single MDA-MB-231 cell by 8 rounds of sampling. The MS/MS spectra of the PB products of PC 32:1, PC 33:1, PC 35:2, PC 36:2, PC 36:1, PC 34:2, and PC 34:1 from a single MDA-MB-231 cell were shown in Supplementary Fig. 4. This demonstrated that detailed lipid structure analysis at the single-cell level could now be performed through chemical derivatization and tandem MS.

### Lipid structural elucidation in single cells

**Fig. 3:**
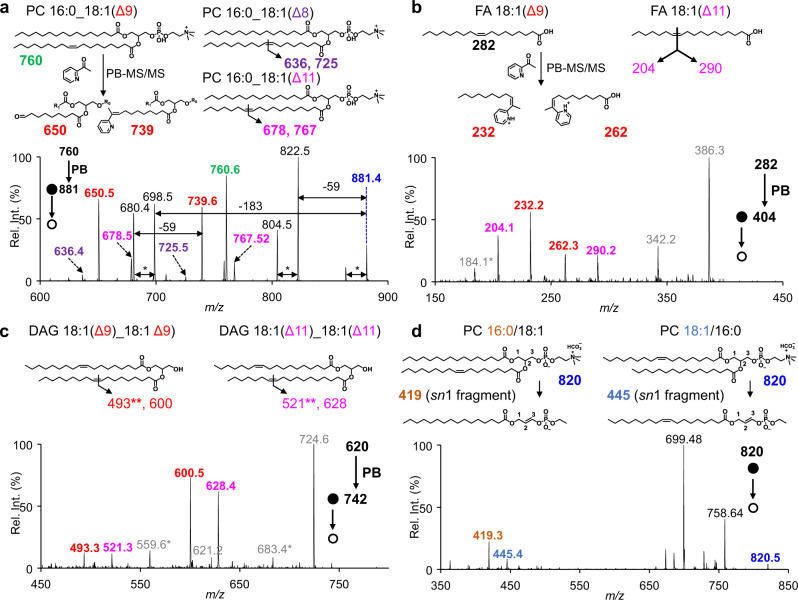
Lipid structural elucidation in single cells. **a** Chemical structure of C=C location and their specific diagnostic ions for PC 16:0_18:1, where the C=C in C18:1 was located at 8, 9, or 11 and the MS/MS spectra of ^PB^(PC 16: 0_18:1) (*m/z* 881.5) from a single MDA-MB-231 cell. The symbol * indicated a loss of H_2_O. **b, c** Chemical structure of C=C location and their specific diagnostic ions for FA 18:1 (**b**) and DAG (18:1_18:1) (**c**), where the C=C in C18:1 was located at 9 or 11 and the corresponding MS/MS spectra of ^PB^(FA 18:1) (*m/z* 282.3) and ^PB^(DAG 18:1_18:1) (*m/z* 620.5) from a single MCF-7 cell. *Fragment ions characteristic to PCs (*m/z* 184 and the signals due to neutral losses of 59 and 183 Da) are detected alongside with FA and DAG. This can be explained by the co-isolation of isobaric PC(s) to the lipid of interests. **d** Chemical structure of PC 16:0/18:1 and PC 18:1/16:0, their diagnostic ions generated by negative ion mode MS/MS analysis with the adduct of bicarbonate anion and the MS/MS spectra of [PC 34:1 + HCO_3_]^−^ (*m/z* 820) in a single MDA-MB-231 cell. All diagnostic ions in the MS/MS spectrum are labeled with the same color as in the corresponding chemical structures.

Coupling with tandem MS, the C=C location(s) in unsaturated lipids could be readily identified. For instance, the pair of diagnostic ions at *m/z* 650 and *m/z* 739 were used to pinpoint a Δ9 C=C in PC 16:0_18:1 (*m/z* 760), following collision-induced dissociation (CID) of the corresponding products (Supplementary Fig. 5). Three C=C location isomers could be detected for PC 16:0_18:1, with the C=C in C18:1 at Δ8, Δ9, and Δ11 as confirmed by diagnostic ions at *m/z* 636/725, 650/739, and 678/767, respectively (Fig. 3a). Moreover, the relative intensities of these C=C-specific diagnostic ions allowed relative quantitation of the corresponding lipid C=C location isomers.

Prior to locating the C=C bond in a fatty acyl-chain, negative MS/MS analysis of [PC + CH_3_COO]^−^ ions from the extract of MDA-MB-231 cells was performed to identify the two fatty acyls^34^. For instance, negative MS/MS analysis of [PC 34:1 + CH_3_COO]^−^ (*m/z* 818) in MDA-MB-231 cell extract showed that the unsaturated fatty acyl in PC 34:1 was C18:1 (major) or C16:1 (minor) (Supplementary Fig. 6a–c). It should be noted that these diagnostic ions could not be used for relative quantitation due to their varied collision efficiencies^52^. These negative MS/MS experiments were also performed at the single-cell level and the results of the major fatty acyl-chain compositions of the lipids in single cells were identical to those in the cell extract (Supplementary Fig. 6d–i).

MS/MS analysis of the corresponding PB products, for example [^PB^(PC 34:1) + H]^+^ (*m/z* 881), pinpointed the C=C location in C18:1 from PC 34:1 to be at Δ8, Δ9, or Δ11 (Fig. 3a). Similarly, MS/MS spectra of [^PB^(PC 36:2) + H]^+^ (*m/z* 907) indicated that unsaturated fatty acyls in PC 36:2 included C18:1(Δ8), C18:1(Δ9), C18:1(Δ11), and C18:2(Δ9, 12) (Supplementary Fig. 7). Other major fragments in Fig. 3a (*m/z* 822, 698, 680) were mainly fragments after neutral losses, for instance, of choline (59 Da) and PC headgroup (183 Da). The lowest abundance lipid detected with C=C locations was PC 38:2 (*m/z* 814) (Supplementary Fig. 10), whose intensity was ~5% of that of PC 34:1 (*m/z* 760.5) (Fig. 1c) and lipid with highest degree of unsaturation was PC 38:5 with 22 diagnostic ions (Supplementary Fig. 8). It is worth mentioning that the relative intensities of diagnostic ions acquired using in-trap CID were much higher than those acquired using beam-type CID (Supplementary Fig. 9). Therefore, all MS/MS analyses of PB products in this study were performed using in-trap CID unless otherwise stated. From single MDA-MB-231 cells, a total of at least 44 structurally defined molecular lipids with C=C locations from 14 sum compositions could be identified (Supplementary Fig. 10 and Supplementary Tables 1 and 2). For each individual cell, around 30 lipid C=C location isomers from 8 sum compositions could be detected by 8 rounds of sampling.

Besides PCs, other classes of lipids were also successfully detected from single cells, including fatty acids (FAs), diacylglycerols (DAGs), triacylglycerols (TAGs), and cholesteryl esters (CEs). FAs and neutral lipids, such as DAGs, TAGs, and CEs, were usually difficult to analyze directly in the positive ion mode. To our surprise, after derivatization these lipids became detectable in positive ion mode, owing to the introduction of a pyridine in PB products. For example, in single MCF-7 cells, positive MS/MS analysis of [^PB^(FA 18:1) + H]^+^ (*m/z* 404) revealed the C=C location in FA 18:1 to be at Δ9 or Δ11, with the two pairs of diagnostic ions at *m/z* 232/262 and 204/290 (Fig. 3b). Positive MS/MS analysis of [^PB^(DAG 18:1_18:1) + H]^+^ (*m/z* 742) indicated that C18:1 within DAG 18:1_18:1 could be either C18:1(Δ9) or C18:1(Δ11), with two corresponding pairs of diagnostic ions at *m/z* 493/600 and 521/628 (Fig. 3c). Positive MS/MS analysis of [^PB^(TAG 18:1_18:1_18:1) + H]^+^ (*m/z* 1006) indicated that C18:1 in TAG 18:1_18:1_18:1 could be either C18:1(Δ9) or C18:1(Δ11), with the two pairs of diagnostic ions at *m/z* 493/864 and 521/892 (Supplementary Fig. 11a). The diagnostic ions containing aldehyde observed for DAGs or TAGs were generated by a concomitant loss of water or a fatty acyl, respectively, after C=C cleavage. The MS/MS spectra of [^PB^(CE 18:1) + H]^+^ (*m/z* 772) indicated that the C=C locations in CE 18:1 were Δ9 or Δ11 with diagnostic ions at *m/z* 630 and 658 (Supplementary Fig. 11b). The characteristic fragment ion of CEs at *m/z* 369 was detected, which confirmed their presence^53^. All these results demonstrated the ability of single-cell PB-MS/MS analysis for a variety of lipid classes.

In addition to the C=C locations, *sn*-positions of PCs in single cells could also be identified. It has been reported that the *sn*-position of PCs could be identified by negative mode MS/MS analysis with the adduct of bicarbonate anion (HCO_3_^−^)^25^. This method was adapted in this study for the single-cell lipid analysis by changing the assisted solvent to 50 mM ammonium bicarbonate solvent in methanol:acetonitrile:water = 4.5:4.5:1 (v/v). The bicarbonate anion was bound strongly with the phosphocholine head group and thus facilitated homolytic bond cleavage (C–N bond) and formation of radical ions upon CID, which generated diagnostic ions critical for identification of *sn*-isomers. As shown in Fig. 3d, two diagnostic ions with the *m/z* of 419 and 445 were detected, corresponding to PC 16:0/18:1 and PC 18:1/16:0, respectively. It should be noted that due to the lipid species limit of this methods we could only analyze the *sn*-position isomeric compositions of PCs in single cells. In summary, the developed protocol is enabling specificity of both C=C locations or *sn*-positions for single-cell lipidomic analysis.

### Comparisons of the degree of cellular heterogeneities in human breast cancer cell lines

Breast cancer is the most frequent malignancy in women and is a heterogeneous disease at the cellular and molecular levels^54^. To explore the lipidome heterogeneity at detailed-structure level among different breast cancer subtypes, other cell lines of MDA-MB-468, MCF-7 and BT-474 were also analyzed by the developed method. MDA-MB-231 and MDA-MB-468 were classified as triple-negative breast cancer cells, while MCF-7 and BT-474 were Luminal A type and Luminal B type, respectively^55^. All the cell lines were subjected to the same process with cell fixation and lipid derivatization, and then analysis by MS (Supplementary Fig. 12). It is worth noting that in single intact MCF-7 cells, peaks isobaric to PB products of major PCs were detected, which were assigned to be TAGs via MS/MS analysis (Supplementary Fig. 13). Some odd-chain PCs were observed, which were consistent with previously published results for these cells^56,57^. Typical MS/MS spectra of [PCs + CH_3_COO]^−^ in negative ion mode for these cells were shown in Supplementary Fig. 14. After PB-MS/MS analysis, tens of structurally defined molecular lipids with C=C location(s) specified were detected in each cell line (Table 1, Supplementary Fig. 15, and Supplementary Tables 3–8). To quantify the relative amounts of lipid C=C location isomers in different breast cancer cell lines, five PCs of high abundances, i.e., PC 32:1, PC 34:2, PC 34:1, PC 36:2, and PC 36:1, were analyzed for ~40 single cells of each subtype.

**Table 1:** The number of lipid isomers detected in single cells of different cell lines.

Cell lines	Sum compositions	Structurally defined molecular lipids with C=C locations
MDA-MB-468	11	37
MCF-7	12	35
MDA-MB-231	14	44
BT-474	11	42

**Fig. 4:**
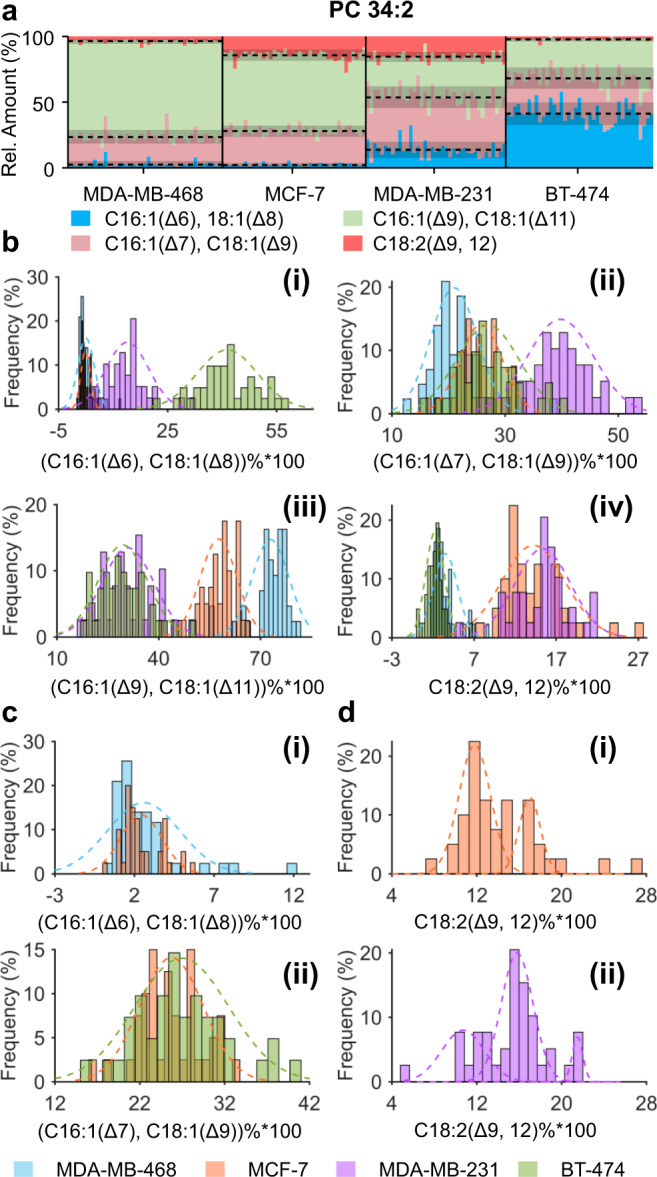
Relative abundance of structurally defined molecular lipids with C=C location specificity in four different subtypes of human breast cancer cell lines. **a–d** Overview (**a**) and frequency histogram (**b**) of relative abundance of C=C location isomers of PC 34:2 in four types of single cells. **c** Comparisons of the relative abundance of PC 34:2 with C16:1(Δ6) or C18:1(Δ8) between MDA-MB-468 and MCF-7 single cells (i) and PC 34:2 with C16:1(Δ7) or C18:1(Δ9) between BT-474 and MCF-7 single cells (ii). **d** Multi-peaks fitting of the relative abundance of PC 16:0_18:2(Δ9, 12) in single MCF-7 cells (i) and MDA-231 cells (ii). The numbers (*n*) of single cells analyzed are 43 for MDA-MB-468, 40 for MCF-7, 39 for MDA-MB-231, and 41 for BT-474. Source data are provided in a Source Data file.

The relative amounts of C=C location isomers for each PC were shown in Fig. 4 and Supplementary Fig. 16, some of which were different among different cell lines (*P*-values in Supplementary Table 9). A *t*-distributed stochastic neighbor embedding (*t*-SNE) calculation allowed for successful classification of these four cell lines (Supplementary Fig. 17a).Cells analyzed were chosen stochastically, and such random sampling is speculated to reflect the whole cell population. For validation, we randomly selected two cell subsets containing 25 or 30 cells (Supplementary Fig. 17b, c) from each breast cancer cell line. The cell classification is also successful, indicating that such a sample size can be used to represent the whole population. Interestingly, the triple-negative breast cancer cell lines of MDA-MB-231 and MDA-MB-468 have completely different lipid composition in terms of C=C location and fatty acyl isomers, possibly due to genomic differences^58^.

Remarkably, the means of the relative abundance of PC 34:2 with C16:1(Δ6) or C18:1(Δ8) in MDA-MB-468 and MCF-7 cells showed no significant statistical difference (*P* > 0.05, determined by two-tailed *t*-test) but the variances showed significant statistical difference (*P* < 0.001, determined by *F*-test). The variances could be used to test the degree of heterogeneity among single cells^59^. As shown in Fig. 4c(i), the heterogeneity of the relative abundance of PC 34:2 with C16:1(Δ6) or C18:1(Δ8) in MDA-MB-468 single cells was higher than that in MCF-7 single cells. Same results were also observed in PC 32:1 and PC 36:2 with C16:1(Δ6) or C18:1(Δ8) (Supplementary Fig. 16). Similarly, the heterogeneity of the relative abundance of PC 34:2 with C16:1(Δ7) or C18:1(Δ9) in BT-474 single cells was higher than that in MCF-7 single cells (Fig. 4c(ii)). The heterogeneity of the relative abundance of PC 32:1 with C16:1(Δ7) or C18:1(Δ9) in BT-474 single cells was higher than that in MDA-MB-468 single cells (Supplementary Fig. 16). Besides, the mean and variance of the relative abundance of PC 16:0_18:2(Δ9, 12) showed no significant statistical difference (*P* > 0.05, mean determined by two-tailed *t*-test and variance determined by *F*-test) in MCF-7 and MDA-MB-231 single cells but their frequency distribution was quite different (Fig. 4d).

Subpopulation division could be used to test the heterogeneity among single cells^60^. By multiple peaks fitting of the frequency histogram, we have revealed the variation of cell subpopulations. The relative abundance of the highest frequency in MCF-7 single cells was ~11.8% while that in MDA-MB-231 single cells was ~15.6% (Fig. 4d). The response of cellular lipidome to stearoyl-CoA desaturase (SCD1) inhibition of MDA-Mb-468 cell line was also studied at the single-cell level, demonstrating the capability of developed method to monitor the change of lipids at C=C location level (Supplementary Note 1). All these results demonstrated the capability of the developed method to probe the heterogeneities among single cells which were absent in cell population analysis.

In addition, we also performed relative quantitation of pairwise lipid *sn*-position isomers for the four breast cancer cell lines. It should be noted that negative mode MS/MS analysis of [PC + HCO_3_]^−^ ions was only used for relative quantitation of lipid *sn*-position isomers with the same fatty acyl composition, excluding lipid fatty acyl isomers. We analyzed six pairs of lipid *sn*-position isomers (Supplementary Table 11) in the four breast cancer cell lines and the results (Supplementary Fig. 20) showed that some lipid *sn*-position isomers showed significant differences among these different subtypes (*P*-values in Supplementary Table 12). The relative abundance of lipid *sn*-position isomers could also be used to probe the degree of heterogeneity among single cells. For example, while the means of the relative abundance of PC 16:1/18:1 in MDA-MB-468 and BT-474 cells showed no significant differences (*P* > 0.05, determined by two-tailed *t*-test), the variances showed significant statistical difference (*P* < 0.001, determined by *F*-test), indicating a higher degree of heterogeneity among MDA-MB-468 cells (Supplementary Fig. 21a).The mean and variance of the relative abundance of PC 16:1/18:0 showed no significant statistical difference in MCF-7 and MDA-MB-468 (*P* > 0.05, mean determined by two-tailed *t*-test and variance determined by *F*-test). However, their frequency distributions were quite different, unimodal for MDA-MB-468 cells and bimodal for MCF-7 cells (Supplementary Fig. 21b). Moreover, *t*-SNE results calculated by relative quantitation of lipid *sn*-position isomers showed a lower degree of cell discrimination, compared with that using lipid C=C location isomers (Supplementary Fig. 17). These results indicated that the quantitative analysis of lipid C=C isomers allows a better discrimination of human breast cancer cells.

### Single-cell lipidomics to discriminate drug-resistant cells from wild-type lung cell population

Lung cancer is the leading type of cancer-associated mortality worldwide and non-small-cell lung cancer (NSCLC) is the most common histological type of lung cancer^61^. As an epidermal growth factor receptor (EGFR)-tyrosine kinase inhibitor, gefitinib is widely used as targeted therapy of NSCLC patients, which significantly improves the overall survival rate of lung cancer^62^. However, some patients who initially respond well to gefitinib would develop acquired resistance^63^. The discrimination of gefitinib-resistant and gefitinib-sensitive cells in NSCLC cell population was therefore very important for the precise treatment of NSCLC patients.

**Fig. 5:**
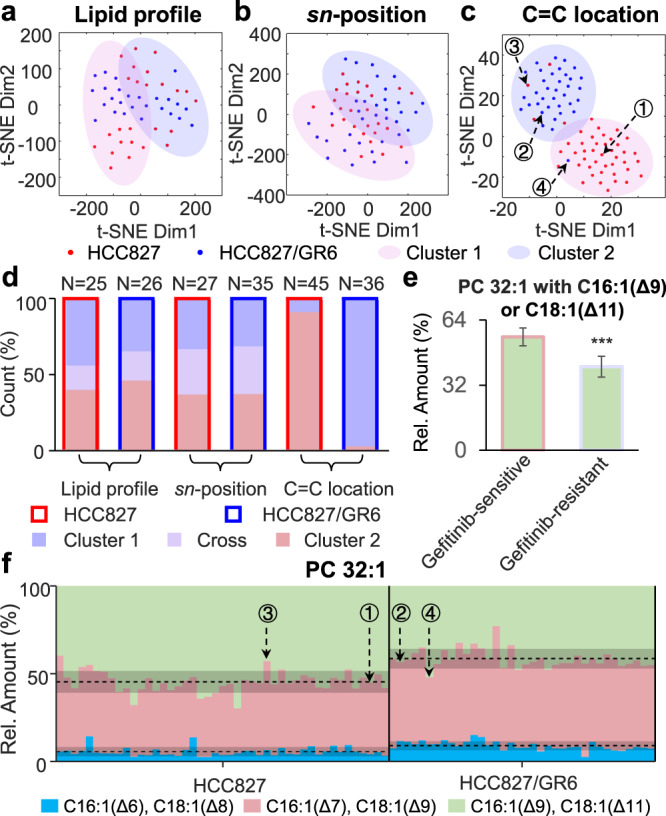
Discrimination of gefitinib-sensitive and gefitinib-resistant human lung cancer cells. **a**–**c** *t*-SNE plot of HCC827 and HCC827/GR6 single cells calculated by molecular lipids profile (**a**), *sn*-position isomer compositions (**b**), or C=C location isomer compositions (**c**). Two cluster areas were circled by k-means clustering results with a confidence probability of 0.95. **d** Cell number distribution in each cluster. **e** Comparison of the relative abundance of PC 32:1 with C16:1(Δ9) or C18:1(Δ11) in gefitinib-sensitive and gefitinib-resistant cells at cell population level. Error bar represents the standard deviation, *n* = 42 for gefitinib-sensitive cells and *n* = 39 for gefitinib-resistant cells. ^***^P < 0.001, determined by single-tailed *t*-test. **f** Relative abundance of C=C location isomers of PC 32:1 in HCC827 and HCC827/GR6 single cells. Source data are provided in a Source Data file.

To explore whether lipid relative quantitation can be used to discriminate gefitinib-resistant and gefitinib-sensitive cells, which coexist in the wild-type NSCLC cell population, we chose two cell lines of HCC827 and HCC827/GR6 for analysis. HCC827 is a wild-type NSCLC cell line and HCC827/GR6 is a gefitinib-resistant cell line inherited from HCC827^64^. The two cell lines were analyzed at the lipid profile, *sn*-position, and C=C location levels. Since lipids with both sodium and proton adduct were observed when 1% formic acid in methanol/acetonitrile = 1:1 (v/v) was used as the assisted solvent, which is undesirable for lipid relative quantitation, 10 mM LiCl in methanol/acetonitrile = 1:1 (v/v) was used instead to convert lipids to their lithium adducts^65^. Twenty-seven lipid sum compositions were analyzed and some of them showed significant differences between the two cell lines (Supplementary Fig. 22 and *P*-values in Supplementary Table 13). However, after *t*-SNE calculation and unsupervised clustering (k-means), 16% of HCC827 and 19% of HCC827/GR6 cells were located in the cross-area of the two clusters, indicating a failed cell classification (Fig. 5a, d).

Lipid analysis at the *sn*-position level was also performed and only one pair of lipid *sn*-isomers (PC 16:1/18:0 and PC18:0/16:1) showed significant differences (Supplementary Fig. 23 and *P*-values in Supplementary Table 14). In the *t*-SNE plot, around 30% of the cells were located in the cross-area (Fig. 5b, d), also indicating a failed cell classification. Finally, we performed lipid analysis at the C=C location level and most of the 17 C=C location isomers detected from 5 lipid sum compositions (Supplementary Table 15) showed significant differences between HCC827 and HCC827/GR6 (Supplementary Fig. 24 and *P*-values in Supplementary Table 16). In the *t*-SNE plot, no cells exist in the cross-area (Fig. 5c, d), indicating a successful cell classification. Since ~98% of cells in Cluster 1 were from the HCC827 cell line and ~90% of cells in Cluster 2 were from the HCC827/GR6 cell line, we hypothesized that cells in Cluster 1 are gefitinib-sensitive while those in Cluster 2 are gefitinib-resistant. Remarkably, ~9% cells from the HCC827 cell line were determined as gefitinib-resistant, highly consistent with the reported percentages (7~12%) of gefitinib-resistant cells in wild-type HCC827 cells^63,66^.

To discover potential biomarkers for accurate discrimination of gefitinib-resistant and gefitinib-sensitive cells, four single cells were selected to compare their lipid C=C location isomer compositions, i.e., Cell #1: HCC827, gefitinib-sensitive; Cell #2: HCC827/GR6, gefitinib-resistant; Cell #3: HCC827, gefitinib-resistant; and Cell #4: HCC827/GR6, gefitinib-sensitive (Fig. 5c, f). At the cell population level, the relative abundance of PC 32:1 with C16:1(Δ9) or C18:1(Δ11) in gefitinib-sensitive cells (55.7 ± 4.9%) was higher than that (41.0 ± 5.1%) in gefitinib-resistant cells (*P* < 0.001, determined by single-tailed *t*-test) (Fig. 5e). Cells #1 and #3 were both from HCC827 cell line: Cell #1 had a proportion of 55.1% of PC 32:1 with C16:1(Δ9) or C18:1(Δ11) and was classified as gefitinib-sensitive, while Cell #3 has a proportion of 42.7% and was classified as gefitinib-resistant. Similarly, Cells #2 and #4 were both from the HCC827/GR6 cell line: Cell #2 has a proportion of 43.4% and was classified as gefitinib-resistant, while Cell #4 has a proportion of 52.1% and was classified as gefitinib-sensitive. Such differences in the relative amounts of PC 32:1 C=C location isomers between gefitinib-resistant and gefitinib-sensitive cells can be readily seen from the PB-MS/MS spectrum (Supplementary Fig. 25). All these results demonstrated that the highly discriminating capability towards drug-resistant cells from an otherwise homogeneous HCC827 cell population cannot be achieved without single-cell structural lipid analysis at C=C location level.

## Discussion

Through this study, we have demonstrated an effective strategy that allows a shotgun lipidomics analysis of single cells with high structural specificity. The cell fixation confined lipids in the cell, which avoided sample dilution and cross-contamination during cell manipulation and batch derivatization. This also lays the foundation for high-throughput single-cell MS analysis. The combination with DAESI promoted single-cell MS analysis to a higher level with structural specificity of the lipidome as well as with high flexibility and sensitivity. From the lipid isomer level, we compared the heterogeneity degree differences among four types of human breast cancer cell lines. Most importantly, our method can reveal inter-cellular heterogeneities among cells in the same population. Gefitinib-resistant single cells could only be discriminated from wild-type NSCLC cell population by lipid C=C location isomer relative quantitation. We believe that chemical derivatization coupled with tandem MS analysis is not only a feasible routine for the structural characterization of biomolecules in single cells, but also a driving force for single-cell biology, cancer pathology, and precision medicine.

## Methods

### Sample preparation

Lipid standards, including PC 15:0-15:0, PE 15:0-15:0 were purchased from Avanti Polar Lipids, Inc. (Alabaster, AL, USA). Acetylpyridines (including 2-AP, 3-AP, and 4-AP) were purchased from Aladdin Biological Technology Company, Ltd. (Shanghai, China). The low-pressure mercury lamp with UV emission centered around 254 nm (Model No. 80-1057-01) was purchased from BHK Inc. (Seongnam-SI, Korea). Dulbecco’s modified Eagle medium (DMEM), Leibovitz’s L-15 medium, phosphate-buffered saline (PBS) solution (10 mM, pH 7.4), Roswell Park Memorial Institute-1640 medium (RPMI), fetal bovine serum (FBS), and Dulbecco’s phosphate-buffered saline (DPBS) were purchased from Gibco Life Technologies (Carlsbad, CA, USA). CAY10566 was purchased from MedChem Express (Monmouth Junction, NJ, USA).

MCF-7 cells were purchased from National Infrastructure of Cell Line Resource (Beijing, China). BT-474, MDA-MB-468, and MDA-MB-231 were obtained from Shanghai Enzyme Research Biotechnology Co. Ltd. (Shanghai, China). MCF-7 and BT-474 cells were cultured in DMEM and RPMI, respectively. MDA-MB-468 and MDA-MB-231 cells were cultured in Leibovitz’s L-15 medium. All cells were supplemented with 10% FBS and 1% penicillin-streptomycin. MDA-MB-468 and MDA-MB-231 cells were cultured with sealed culturing dish while the rest of cells were cultured in breathable dish at 37 °C in a humidified atmosphere containing 5% CO_2_. Cells were washed with DPBS (2 mL) and passaged by trypsinization when they reached 85−90% confluence. For SCD1 inhibition, MDA-MB-468 cells were treated with 100 nM CAY10566 for 3 days, while the control group was treated with DMSO. HCC827 and HCC827/GR6 cells used in this study are kind gifts from Dr. Pasi A. Jänne (Dana-Farber Cancer Institute, Boston, MA). Cell lines were authenticated via short tandem repeat (STR) testing by Genesky Biopharma Technology (Shanghai, China). Cells were maintained in appropriate culture medium, as suggested by the supplier.

After washing with PBS, cells (~10^6^ cells/mL) were suspended in 2.5% glutaraldehyde in PBS for 20 min in a shaker. Cells were then washed twice. For direct single-cell analysis, cells were resuspended in water and diluted to a concentration of 10^4^ cells/mL. For cell derivatization, cells (~10^6^ cells/mL) were resuspended in 100 mM 2-AP aqueous solution. The device for cell derivatization is shown in Fig. 1c. And after 2 min UV irradiation, the quartz cell was taken out and shaken gently for even cell distribution. This process was repeated 4 times, reaching a reaction time of 8 min. Derivatized cells were washed twice using water to remove excess 2-AP and finally diluted to a concentration of 10^4^ cells/mL. The fixation of the cell population (e.g., 10^6^ cells) requires 20 min, the PB derivatization needs 8 min, and the cell washing using centrifugation requires another 10 min, bringing the total time of cell preparation to around 40 min. These extra steps are necessary to acquire detailed lipid structures, e.g., C=C locations, but performed to the cell population so the time of cell preparation would not increase as the number of cells increases.

A modified Folch method was used for lipid extraction from the cell population. Harvested cells were divided into two equal subsets. One subset was treated with 2.5% glutaraldehyde in PBS for fixation while the other subset was treated with PBS as a control. After centrifugation, cells were resuspended in 1 mL deionized water in a 10 mL centrifuge tube, followed by addition of 1 mL methanol and 2 mL chloroform. The internal standard (PC 15:0/15:0 and PE 15:0/15:0) was added at a concentration of 5 μM. After 5 min vortexing, the mixture was centrifuged at 11,269*g* for 8 min. Then, a second round of lipid extraction was performed. The chloroform layers from the two extractions were finally combined and dried under nitrogen flow. The lipid extract was reconstituted in 500 μL methanol and stored at −20 °C for MS analysis.

The stainless-steel electrode (o.d. = 120 μm) was purchased from HAMILTON Company (Reno, NV, USA) and the borosilicate glass capillaries (i.d. = 0.86 mm, o.d. = 1.5 mm) were purchased from Sutter Instrument (Novato, CA, USA). Borosilicate glass capillaries were washed with 1% formic acid (v/v) in ethanol:water = 1:1 twice, and then pulled to make nanoESI emitters (i.d. of the opening ≈ 5~10 μm) for MS analysis, using a P-1000 micropipette puller (Sutter Instrument, Novato, CA, USA). P-1000 parameters are as follows: HEAT = 535, PULL = 0, VEL = 15, DELAY = 1, Pressure = 600, and Ramp = 530.

### Single-cell MS analysis

Around 0.5 ${\rm{\mu }}{\rm{L}}$ of cell suspension was transferred to a nanospray capillary. A DC voltage of −1.3 kV was applied via a stainless-steel electrode for several seconds to promote electro-migration. After solvent evaporation, a droplet (~50 μL) of assistant solvent (methanol:acetonitrile = 1:1 (v/v) with 1% formic acid) was applied to the capillary tip. Meanwhile, a voltage of +1.8 kV was applied to initiate on-demand MS analysis. For PB-MS/MS analysis, multiple droplets were dropped sequentially to the nanoESI tip to analyze different lipid species. It took 1~2 min to transfer a single cell to the capillary and perform electro-migration, 3~5 min to evaporate the liquid in the tip, and 4~6 min for multi-rounds sampling and data acquisition. For lipid sum compositions analysis, a droplet of 10 mM LiCl in methanol:acetonitrile = 1:1(v/v) and a voltage of +1.5 kV were applied for DAESI. For negative MS/MS analysis to confirm fatty acyl-chains, a droplet of 50 mM ammonium acetate solvent in methanol:acetonitrile:water = 4.5:4.5:1 (v/v) and a voltage of −1.8 kV were applied for DAESI. For *sn*-position identification, a droplet of 50 mM ammonium bicarbonate solvent in methanol:acetonitrile:water = 4.5:4.5:1 (v/v) and a voltage of −1.8 kV were applied for DAESI. All MS analysis was performed by QTRAP 4500 mass spectrometer (Sciex, Toronto, CA).

### Data processing

The recovery ratio of PCs after fixation is defined as follows:

1 $${\mathrm{Recovery}}\,(\%)=\frac{{\mathrm{Int}}({\mathrm{P}}{{\mathrm{C}}}_{{\mathrm{withfixation}}})/{\mathrm{Int}}({\mathrm{I}}{{\mathrm{S}}}_{{\mathrm{withfixation}}})}{{\mathrm{Int}}({\mathrm{P}}{{\mathrm{C}}}_{{\mathrm{withoutfixation}}})/{\mathrm{Int}}({\mathrm{I}}{{\mathrm{S}}}_{{\mathrm{withoutfixation}}})}\times100\%$$

The conversion yield of PB reaction is defined as follows:

2 $${\mathrm{Yield}}=\frac{{\sum }^{\,} {\mathrm{Int}}({\,}^{\mathrm{PB}}({\mathrm{PCs}}))} {\sum {\mathrm{Int}}({\,}^{\mathrm{PB}}({\mathrm{PCs}}))+\sum {\mathrm{Int}}({\mathrm{remaining}}\,{\mathrm{PCs}})}\times 100\%$$

Pairwise correlation coefficients in Fig. 2e and Supplementary Fig. 3 were calculated by the intensities of PB product ions (Supplementary Table 2) of each spray. The intensities of C=C-specific diagnostic ions are used for quantifying lipid C=C location isomers. The S/N ratio of the diagnostic ions should be >10 for quantitation. For a lipid containing *n* C=Cs, its relative amount is represented by the total intensities of all dignostic ions, divided by *n*. Below, we use PC 36:2 as an example to show the calculation,

3 $${\mathrm{C18:1}}({\Delta }8) \% =\frac{\sum {\mathrm{Int}}(662,751)}{\sum{\mathrm{ Int}}(662,751;676,\,765;704,793)+\sum \frac{{\mathrm{Int}}(678,767,718,807)}{2}}$$

4 $${\mathrm{C18:1}}({\Delta }9) \% =\frac{\sum{\mathrm{ Int}}(676,765)}{\sum{\mathrm{ Int}}(662,751;676,765;704,793)+\sum \frac{{\mathrm{Int}}(678,767,718,807)}{2}}$$

5 $${\mathrm{C18:1}}({\Delta }11) \% =\frac{\sum {\mathrm{Int}}(704,793)}{\sum{\mathrm{ Int}}(662,751;676,765;704,793)+\sum \frac{{\mathrm{Int}}(678,767,718,807)}{2}}$$

6 $${\mathrm{C18:1}}({\Delta }9,12) \% =\frac{{\sum }^{}{\mathrm{Int}}(678,767,718,807)/2}{\sum {\mathrm{Int}}(662,751;676,765;704,793)+\sum \frac{{\mathrm{Int}}(678,767,718,807)}{2}}$$

Two-tailed *t*-test was performed to evaluate the changes of the average of relative amounts of PC C=C location isomers and *F*-test was performed to evaluate the changes of the variances. All MS data were collected and processed by Analyst 1.6.3 from Sciex. All data processing including distribution fitting, *t*-test, *F*-test, *t*-SNE calculation, and k-means clustering were performed using MATLAB 2019b.

### Reporting summary

Further information on research design is available in the Nature Research Reporting Summary linked to this article.

## Supplementary information

Supplementary information

Reporting summary

## Data Availability

All data supporting the findings of this study are available from the corresponding authors upon reasonable request. The raw MS data are available from Figshare (10.6084/m9.figshare.14381258.v3). Source data are provided with this paper.

## Source data

Source Data
