# Supplementary material for: Single-cell lipidomics with high structural specificity by mass spectrometry
Source: Nat Commun. 2021 May 17;12:2869. doi: 10.1038/s41467-021-23161-5 (PMC8129106; doi:10.1038/s41467-021-23161-5)
Supplement: Supplementary file 1 — Supplementary information [file 41467_2021_23161_MOESM1_ESM.docx]

**Supplementary Information**

**Single-cell Lipidomics with High Structural Specificity by Shotgun Mass Spectrometry**

Zishuai Li^1^, Simin Cheng^1^, Qiaohong Lin^2^, Wenbo Cao^1^, Jing Yang^1^, Minmin Zhang^4^, Aijun Shen^4^, Wenpeng Zhang^3^, Yu Xia^2,3^, Xiaoxiao Ma^1*^, Zheng Ouyang^1,3*^

**Contents:**

Supplementary Figures 1-24

Supplementary Tables 1-16

Supplementary Note 1


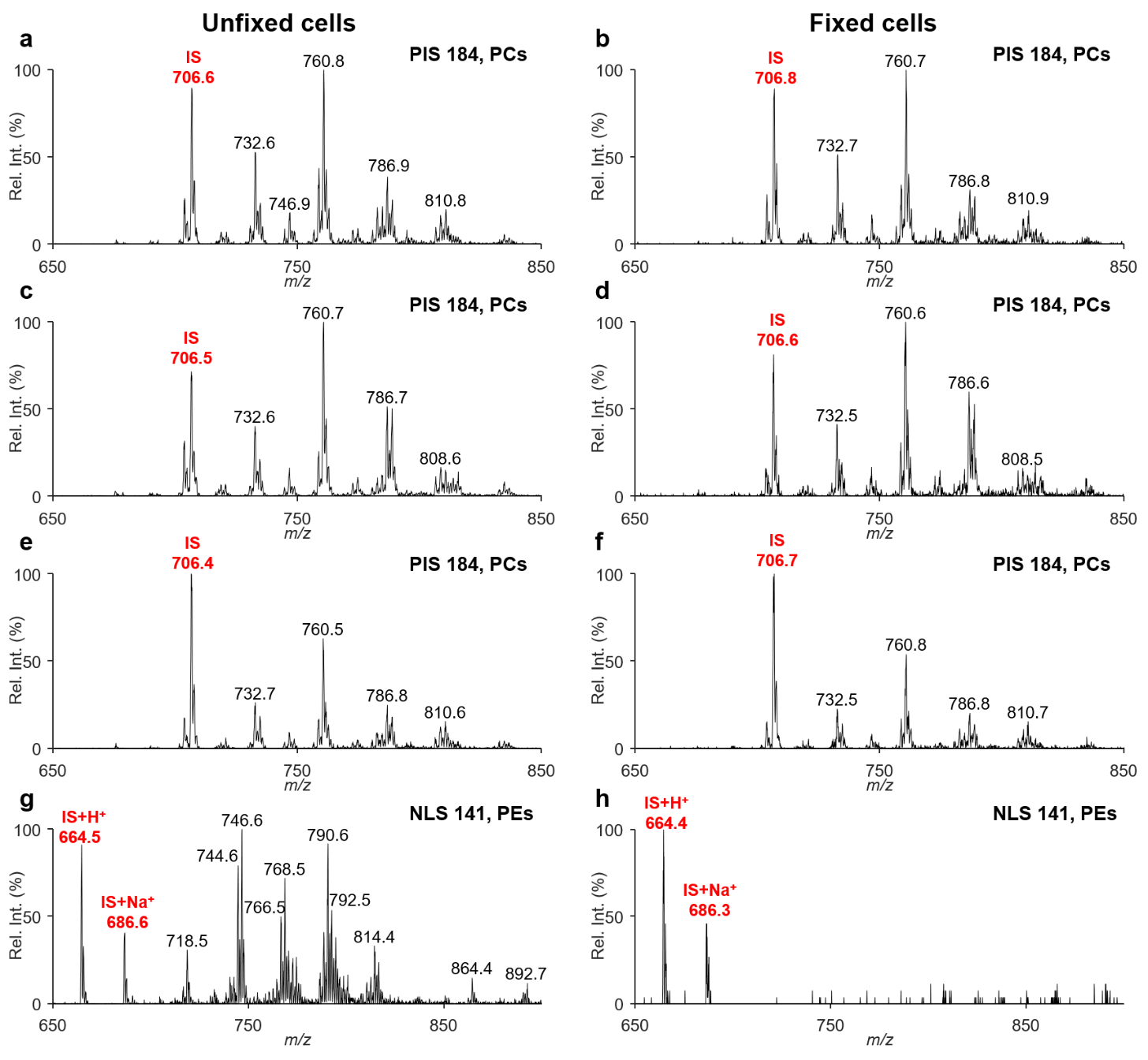


**Supplementary Fig. 1. Lipid profiling in unfixed and fixed cells. (a-f)** Spectrum of precursor ion scan of 184 (PCs) of unfixed (**a, c, e**) and fixed (**b, d, f**) MDA-MB-231 cells. The cell concentration in **a**, **b**, **c**, and **d** was 10^6^ cells/ml and in **e** and **f** was 5x10^5^ cells/ml. Internal standards: PC 15:0-15:0 (*m/z* 706). (**g, h)** Spectra of neutral loss of 141 (PEs) of unfixed (G) and fixed (H) MDA-MB-231 cells. Internal standards (IS): PE 15:0-15:0 ([M+H]^+^ *m/z* 664, [M+Na]^+^ *m/z* 686).


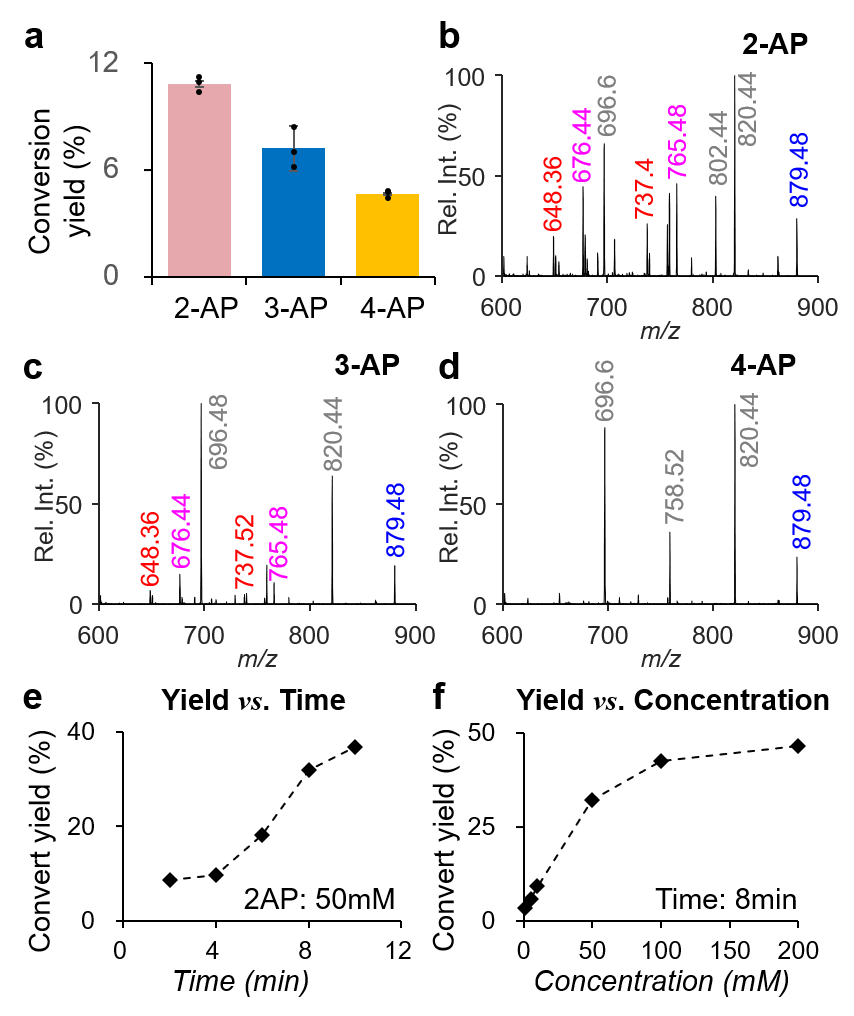


**Supplementary Fig. 2. Experimental optimization of the photochemical derivatization of mammalian cells (MCF-7 cells).** (**a**) The conversion yields using 2-AP, 3-AP and 4-AP. Reaction time: 5 min, photochemical reagent concentration: 50 mM. Error bar represents the standard deviation, n =3. (**b-d)** MS/MS spectra of PC 34:2 after reaction with 2-AP (**b**), 3-AP (c), and 4-AP (**d**). The C=C-specific DIs were at *m/z* 648/737 (Δ9) and 676/765 (Δ11) and labeled with red and pink, respectively. (**e-f)** The conversion yield as a function of reaction time (**e**) or 2-AP concentration (**f**). Source data are provided in a Source Data file.


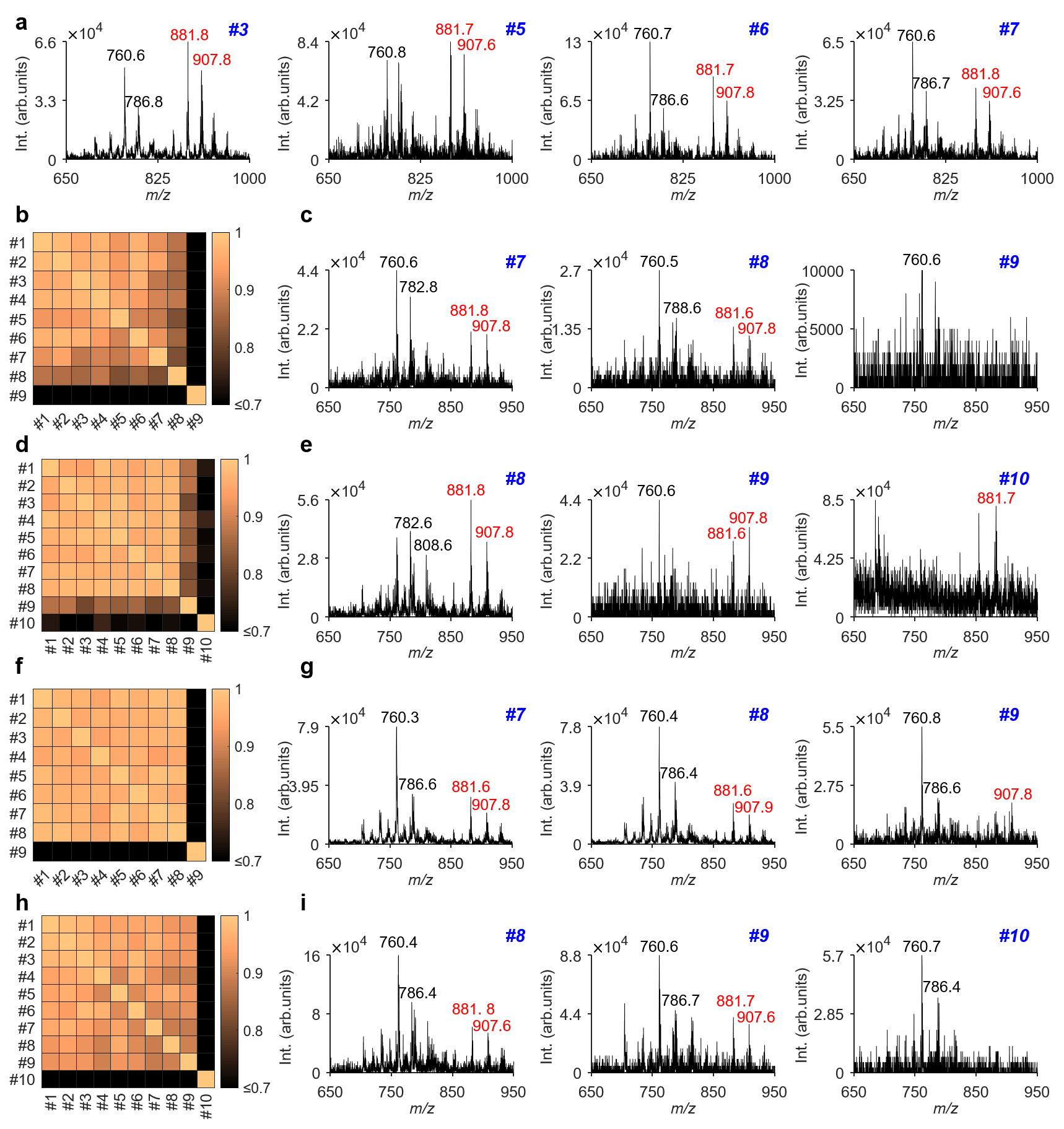


**Supplementary Fig. S3. Reproducibility of on-demand DAESI. (a)** Average MS spectra of the 3^rd^, 5^th^, 6^th^, 7^th^ samplings in Fig. 2C. (**b-i)** Repeat of the experiments. (**b, d, f, h)** Pairwise correlation coefficients calculated by the intensities of PB product ions of each spray. **(c, e, g, i)** Average MS spectra of the last three samplings in each repeat experiment. The peaks of PB product ions are labeled with red.


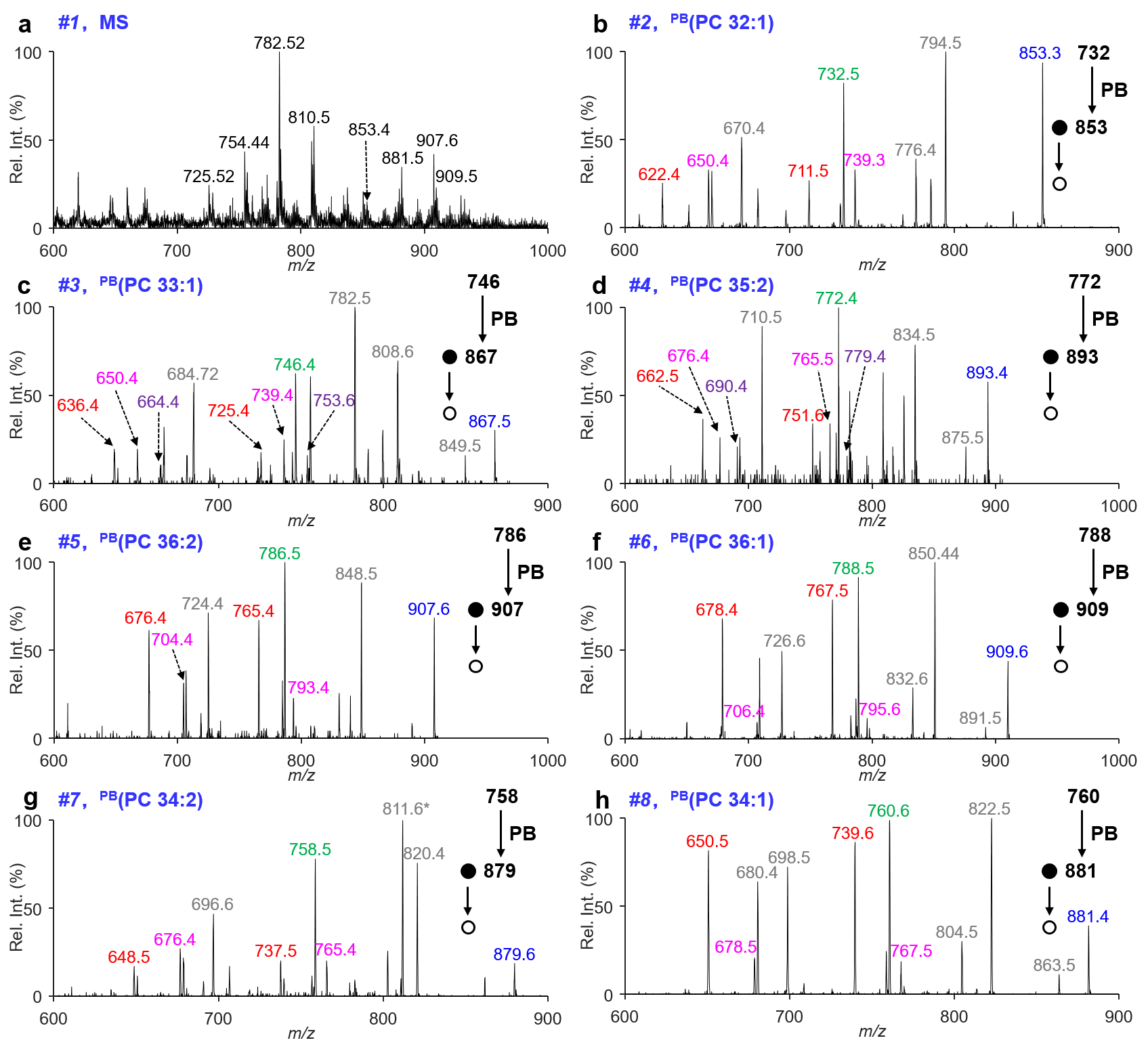


**Supplementary Fig. 4. On-demand MS/MS analysis of derivatized lipids from a single MDA-MB-231 cell.** (**a)** MS1 spectrum, (**b-h)** MS/MS spectra of [^PB^(PC 32:1)+H]^+^ (*m/z* 853), [^PB^(PC 33:1)+H]^+^ (*m/z* 867), [^PB^(PC 35:2)+H]^+^ (*m/z* 893), [^PB^(PC 36:2)+H]^+^ (*m/z* 907), [^PB^(PC 36:1)+H]^+^ (*m/z* 909), [^PB^(PC 34:2)+H]^+^ (*m/z* 879) and [^PB^(PC 34:1)+H]^+^ (*m/z* 881) from a single MDA-MB-231 cell. The diagnostic ions are marked with red or pink for n-7 and n-9 isomers, respectively. The precursor ions are labeled with blue, and the fragment ions with a loss of 2-AP are labeled with green.


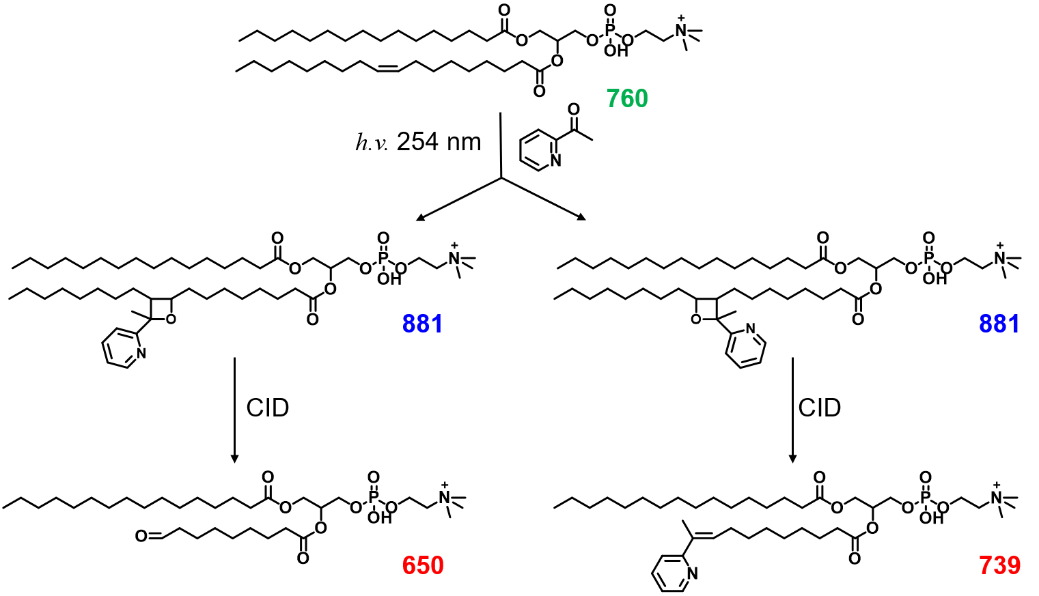


**Supplementary Fig. 5. Scheme for the fragmentation of PB products of PC 16:0_18:1(Δ9).** PC 16:0_18:1(Δ9) (*m/z* 760) was converted to ^PB^(PC 16:0_18:1(Δ9)) (*m/z* 881) as two regio-isomers after PB reaction. The two C=C-specific diagnostic ions, i.e. aldehyde (*m/z* 650) and pyridine (*m/z* 739), were generated from the two regio-isomers.


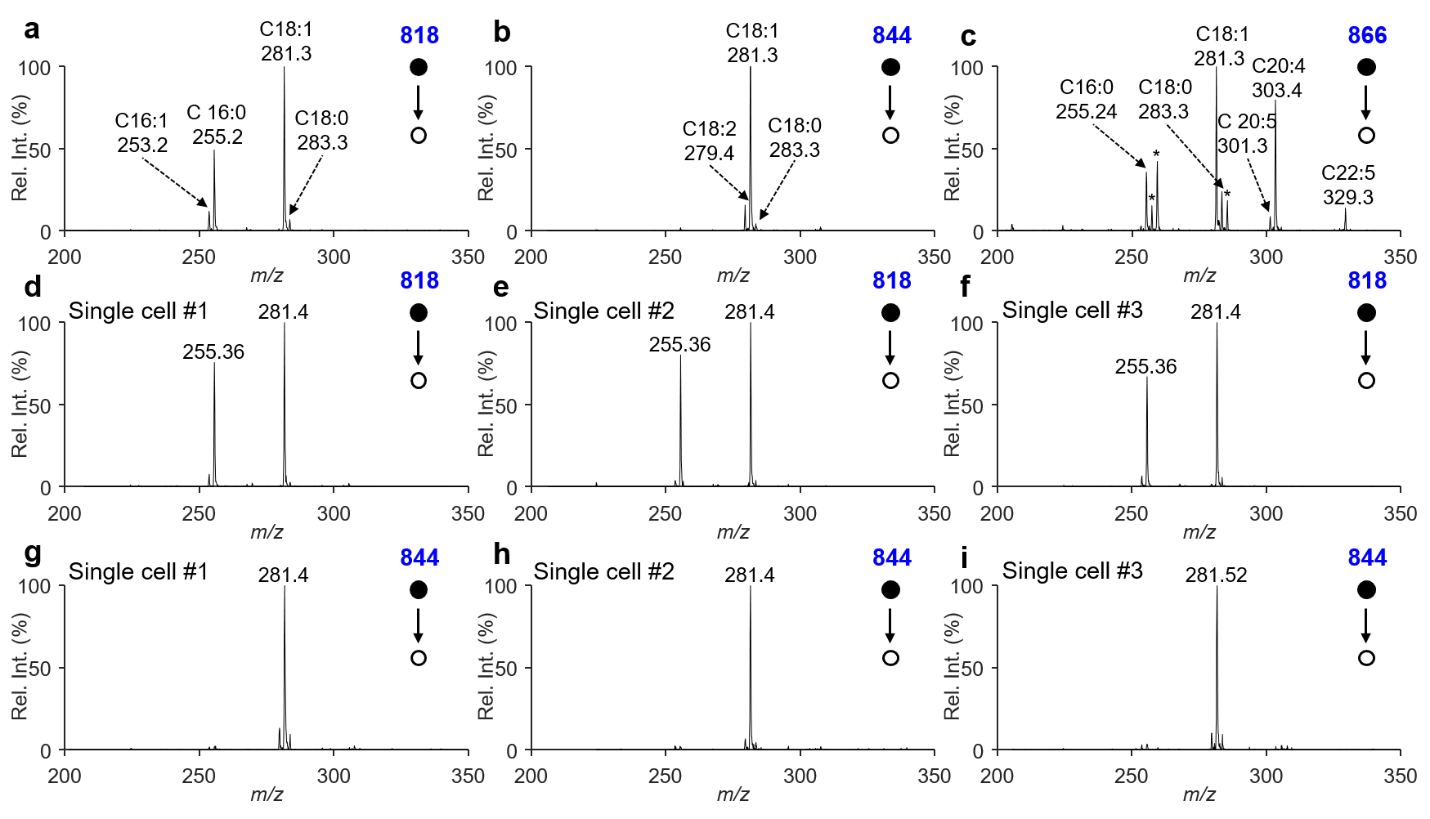


**Supplementary Fig. 6. Negative MS/MS spectra of lipids from the MDA-MB-231 cell extract and MDA-MB-231 single cells. (a-c)** MS/MS spectra of the cell extract. (**a)**: [PC 34:1+CH_3_COO]^-^ (*m/z* 818), **(b)**: [PC 36:2+CH_3_COO]^-^ (*m/z* 844) and **(c)**: [PC 38:5+CH_3_COO]^-^ (*m/z* 866). **(d-i)** Spectra from single cells. **(d-f)** [PC 34:1+CH_3_COO]^-^ (*m/z* 818) from 3 different single cells, **(g-i)** [PC 36:2+CH_3_COO]^-^ (*m/z* 844) from 3 different cells. *A typical neutral loss of 44 Da for polyunsaturated fatty acyls.


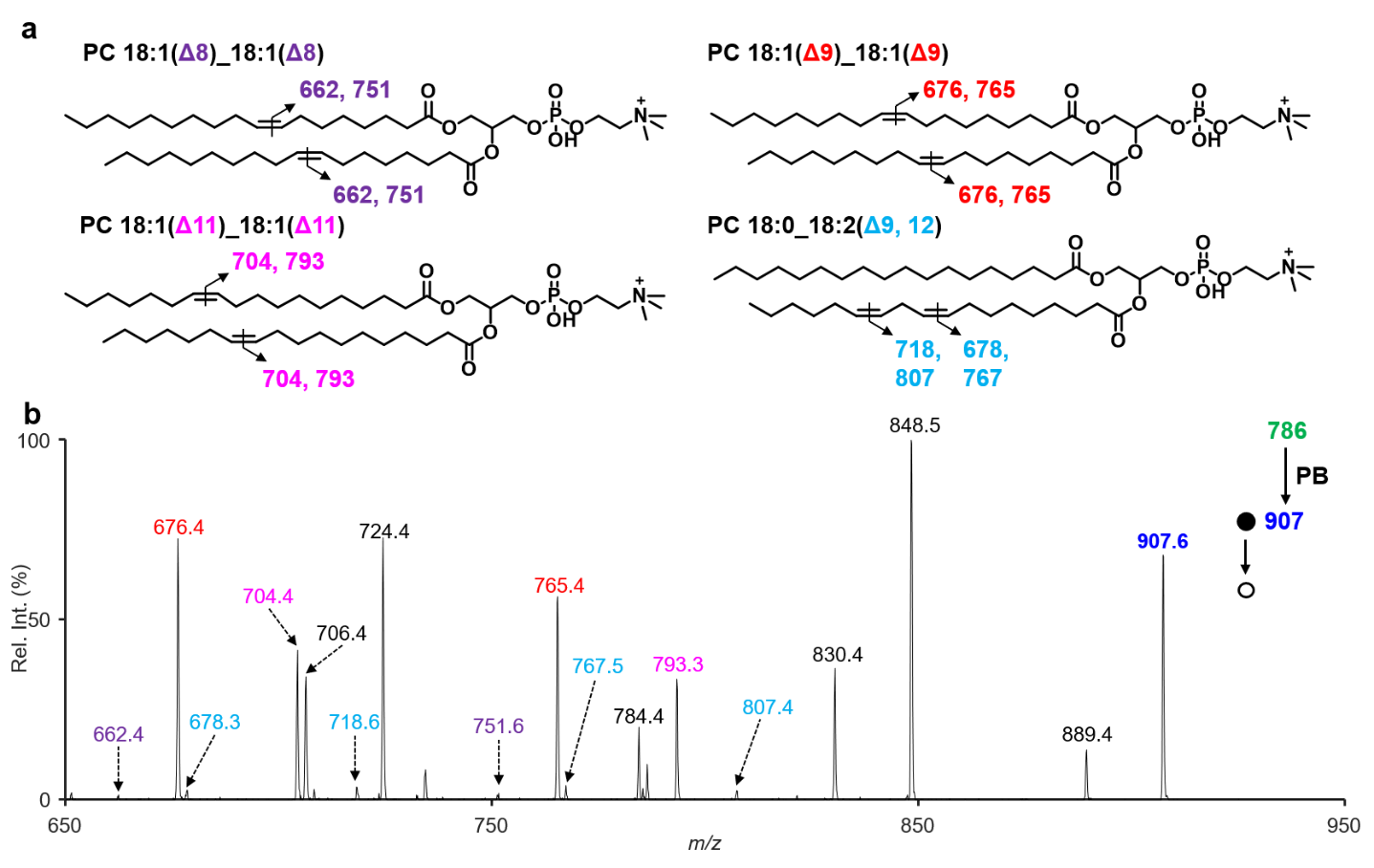


**Supplementary Fig. 7. PB-MS/MS analysis of PC 36:2. (a)** C=C-specific fragmentations and corresponding DIs in PC 36:2. **(b)** MS/MS spectra of [^PB^(PC 36:2)+H]^+^ from a single MDA-MB-231 cell. All diagnostic ions in the MS/MS spectrum are labeled with the same color as in the corresponding chemical structures.


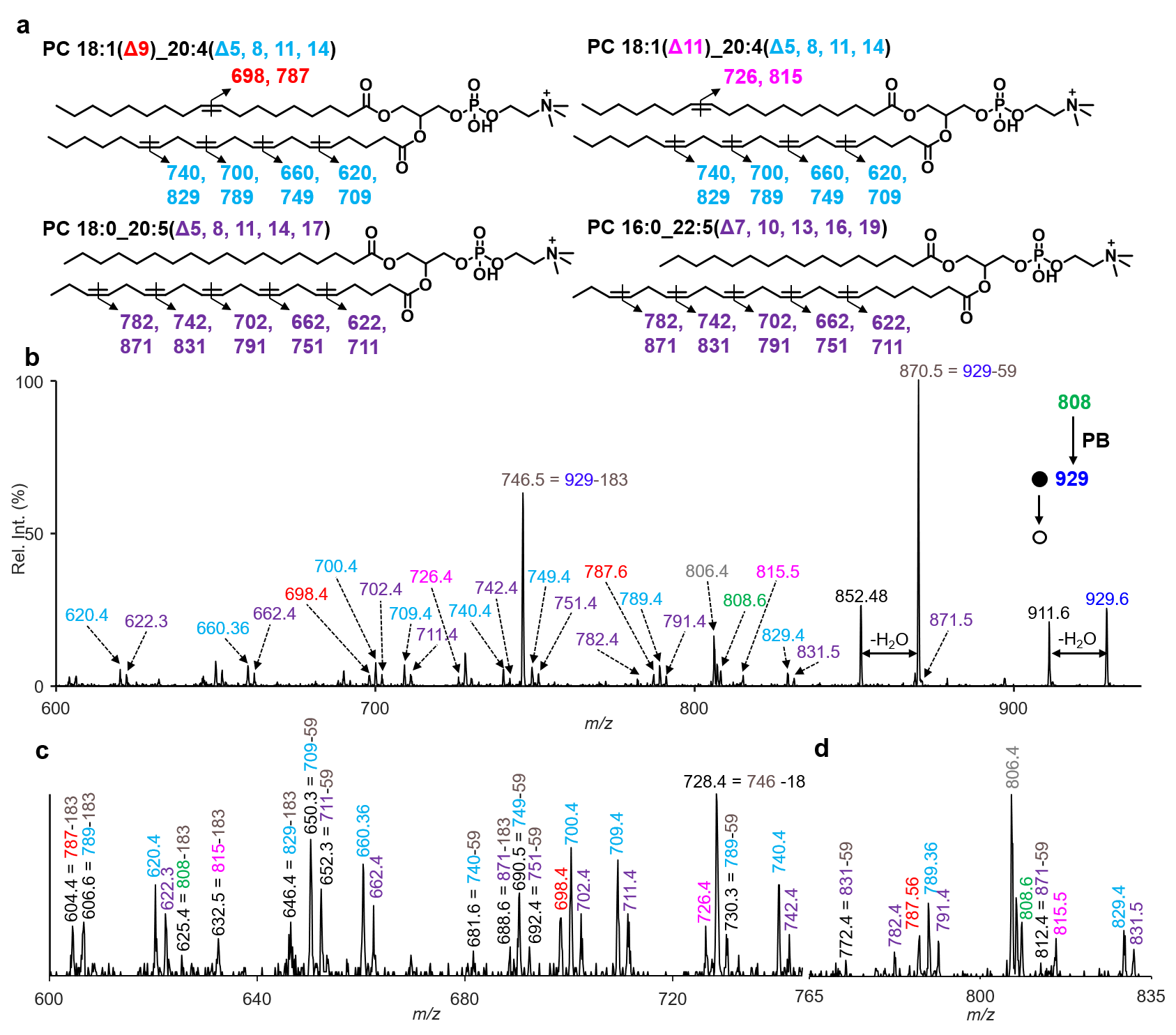


**Supplementary Fig. 8. PB-MS/MS analysis of PC 38:5. (a)** Structure of PC 38:5 with the diagnostic ions of C=C locations. **(b)** MS/MS spectra of ^PB^(PC 38:5) in a single MDA-MB-231 cell. The diagnostic ions were marked with the same color. **(c-d)** Magnified views of the m/z ranges of 600~745 **(c)** and 765~835 **(d)**. The subtraction formula indicated neutral losses of 59 Da (choline), 183 Da (head group) and 18 Da (H_2_O) from diagnostic ion. All diagnostic ions in the MS/MS spectrum are labeled with the same color as in the corresponding chemical structures. All peaks with a S/N ratio of no less than 3 were labelled.


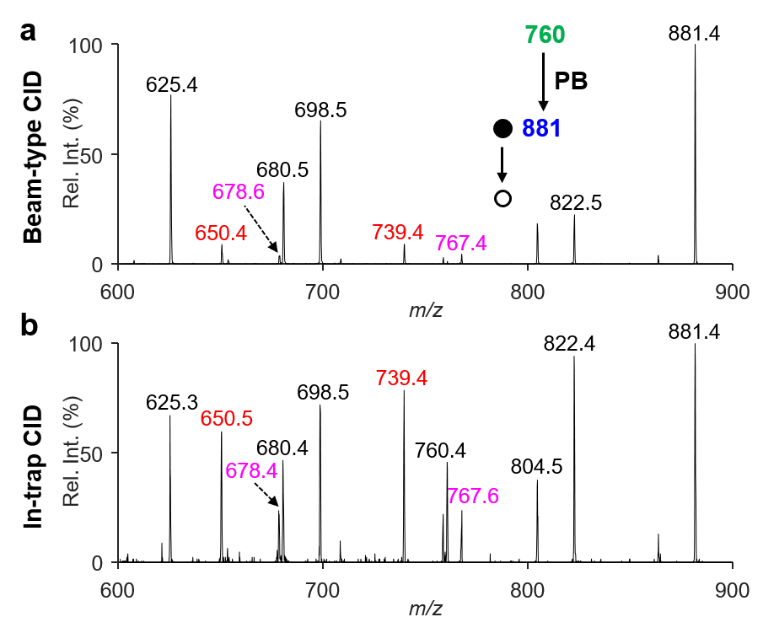


**Supplementary Fig. 9. Comparison of beam-type CID (a) and in-trap CID (b) on AB Sciex QTRAP4500 for the analysis of derivatized lipids, using PC 34:1 from a single MDA-MB-231 cell as an example.** The C=C-specific diagnostic ions are at *m/z* 650/739 (Δ9) and 678/7675 (Δ11) and labeled with red and pink, respectively.


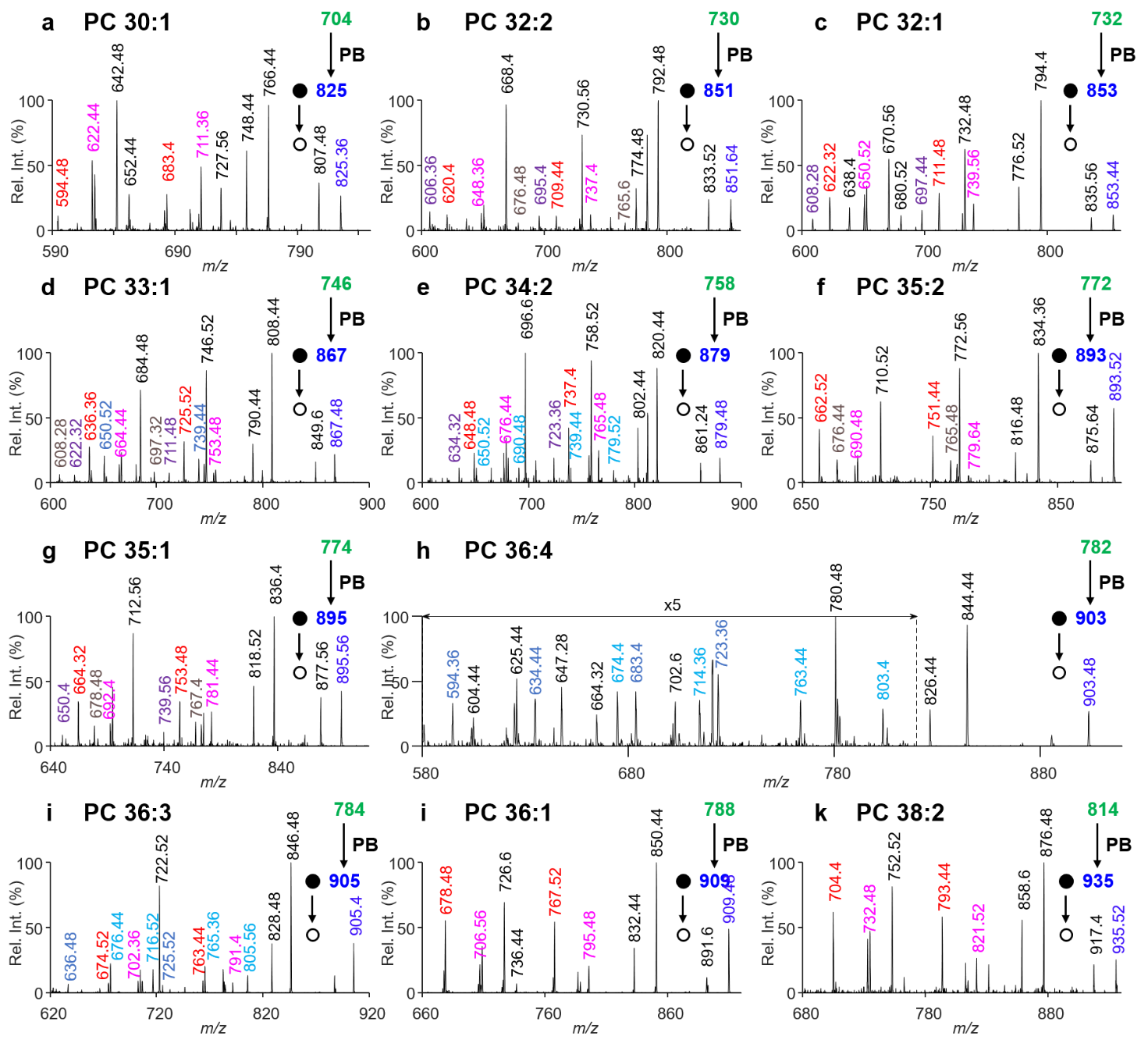


**Supplementary Fig. 10. Tandem MS analysis of derivatized lipids from single MDA-MB-231 cells. (a)** PC 30:1, **(b)** PC 32:2, **(c)** PC 32:1, **(d)** PC 33:1, **(e)** PC 34:2, **(f)** PC 35:2, **(g)** PC 35:1, **(h)** PC 36:4, **(i)** PC 36:3, **(j)** PC 36:1, **(k)** PC 38:2. All the diagnostic ions are marked with colors and are list in Table S2 with isomer assignment.

**Supplementary Table 1.** Fatty acyl chain of lipids in MDA-MB-231 single cells.

| N.O. | m/z | Species | Adduct | Diagnostic Ions | Chain |
| --- | --- | --- | --- | --- | --- |
| 1 | 704.5 | PC 30:1 | 762.5 | 227, 253 | PC 14:0_16:1 |
|  |  |  |  | 225, 255 | PC 14:1_16:0 |
| 2 | 730.5 | PC 32:2 | 788.5 | 253, 253 | PC 16:1_16:1 |
|  |  |  |  | 227, 279 | PC 14:0_18:2 |
|  |  |  |  | 225, 281 | PC 14:1_18:1 |
| 3 | 732.5 | PC 32:1 | 790.5 | 255, 253 | PC 16:0_16:1 |
|  |  |  |  | 227, 281 | PC 14:0_18:1 |
| 4 | 746.5 | PC 33:1 | 804.5 | 255, 267 | PC 16:0_17:1 |
|  |  |  |  | 241, 281 | PC 15:0_18:1 |
|  |  |  |  | 253, 269 | PC 16:1_17:0 |
| 5 | 758.5 | PC 34:2 | 816.5 | 253, 281 | PC 16:1_18:1 |
|  |  |  |  | 255, 279 | PC16:0_18:2 |
| 6 | 760.5 | PC 34:1 | 818.5 | 255, 281 | PC 16:0_18:1 |
|  |  |  |  | 253, 283 | PC 16:1_18:0 |
| 7 | 772.5 | PC 35:2 | 830.5 | 267, 281 | PC 17:1_18:1 |
|  |  |  |  | 269, 279 | PC 17:0_18:2 |
| 8 | 774.5 | PC 35:1 | 832.5 | 269, 281 | PC 17:0_18:1 |
|  |  |  |  | 267, 283 | PC 17:1_18:0 |
|  |  |  |  | 255, 295 | PC 16:0_19:1 |
| 9 | 782.5 | PC 36:4 | 840.5 | 255, 303 | PC 16:0_20:4 |
|  |  |  |  | 279, 279 | PC 18:2_18:2 |
| 10 | 784.5 | PC 36:3 | 842.5 | 281, 279 | PC 18:1_18:2 |
|  |  |  |  | 255, 305 | PC 16:0_20:3 |
| 11 | 786.5 | PC 36:2 | 844.5 | 281, 281 | PC 18:1_18:1 |
|  |  |  |  | 283, 279 | PC 18:0_18:2 |
| 12 | 788.5 | PC 36:1 | 846.5 | 283, 281 | PC 18:0_18:1 |
|  |  |  |  | 255, 309 | PC 16:0_20:1 |
| 13 | 808.5 | PC 38:5 | 866.5 | 281, 303 | PC 18:1_20:4 |
|  |  |  |  | 255, 329 | PC 16:0_22:5 |
|  |  |  |  | 283, 301 | PC 18:0_20:5 |
| 14 | 814.5 | PC 38:2 | 872.5 | 281, 309 | PC 18:1_20:1 |

**Supplementary Table 2.** Lipids C=C location isomers in single MDA-MB-231 cells.

| N.O. | m/z | Species | PB product | Diagnostic Ions | C=C location |
| --- | --- | --- | --- | --- | --- |
| 1 | 704.7 | PC 30:1 | 825.7 | 594, 683 | PC 14:0_16:1(Δ7) |
|  |  |  |  | 622, 711 | PC 14:0_16:1(Δ9) |
| 2 | 730.6 | PC 32:2 | 851.6 | 606, 695 | PC 16:1(Δ6)_16:1(Δ6) |
|  |  |  |  | 620, 709 | PC 16:1(Δ7)_16:1(Δ7) |
|  |  |  |  | 648, 737 | PC 16:1(Δ9)_16:1(Δ9) |
|  |  |  |  | 676, 765; | PC 14:1(Δ9)_18:1 |
| 3 | 732.7 | PC 32:1 | 853.7 | 608, 697 | PC 14:0_18:1(Δ8), PC 16:0_16:1(Δ6) |
|  |  |  |  | 622, 711 | PC 14:0_18:1(Δ9), PC 16:0_16:1(Δ7) |
|  |  |  |  | 650, 739 | PC 14:0_18:1(Δ11), PC 16:0_16:1(Δ9) |
| 4 | 746.6 | PC 33:1 | 867.5 | 608, 697 | PC 16:0_17:1(Δ6) |
|  |  |  |  | 650, 739 | PC 16:0_17:1(Δ9) |
|  |  |  |  | 622, 711 | PC 15:0_18:1(Δ8), PC 16:1(Δ6)_17:0 |
|  |  |  |  | 636, 725 | PC 15:0_18:1(Δ9), PC 16:1(Δ7)_17:0 |
|  |  |  |  | 664, 753 | PC 15:0_18:1(Δ11), PC 16:1(Δ9)_17:0 |
| 5 | 758.7 | PC 34:2 | 879.7 | 634, 723 | PC 16:1(Δ6)_18:1(Δ8) |
|  |  |  |  | 648, 737 | PC 16:1(Δ7)_18:1(Δ9) |
|  |  |  |  | 676, 765 | PC 16:1(Δ9)_18:1(Δ11) |
|  |  |  |  | 650, 739; 690, 779 | PC 16:0_18:2(Δ9, 12) |
| 6 | 760.7 | PC 34:1 | 881.7 | 636, 725 | PC 16:0_18:1(Δ8), PC 16:1(Δ6)_18:0 |
|  |  |  |  | 650, 739 | PC 16:0_18:1(Δ9), PC 16:1(Δ7)_18:0 |
|  |  |  |  | 678, 767 | PC 16:0_18:1(Δ11), PC 16:1(Δ9)_18:0 |
| 7 | 772.5 | PC 35:2 | 893.5 | 676, 765 | PC 17:1(Δ9)_18:1(Δ8) |
|  |  |  |  | 676, 765; 662, 751 | PC 17:1(Δ9)_18:1(Δ9) |
|  |  |  |  | 676, 765; 690, 779 | PC 17:1(Δ9)_18:1(Δ11) |
| 8 | 774.5 | PC 35:1 | 895.4 | 650, 739 | PC 17:0_18:1(Δ8), PC 16:0_19:1(Δ9) |
|  |  |  |  | 664, 753 | PC 17:0_18:1(Δ9) |
|  |  |  |  | 692, 781 | PC 17:0_18:1(Δ11) |
|  |  |  |  | 678, 767 | PC 17:1(Δ9)_18:0 |
| 9 | 782.6 | PC 36:4 | 903.6 | 594, 683; 634, 723; 674, 763; 714, 803 | PC 16:0_20:4(Δ5, 8, 11, 14) |
|  |  |  |  | 674, 763; 714, 803 | PC 18:2(Δ9, 12)_18:2(Δ9, 12) |
| 10 | 784.7 | PC 36:3 | 905.7 | 636, 725; 676, 765; 716, 805 | PC 16:0_20:3(Δ8, 11, 14) |
|  |  |  |  | 674, 763; 676, 765; 716, 805 | PC 18:1(Δ9)_18:2(Δ9, 12) |
|  |  |  |  | 702, 791; 676, 765; 716, 805 | PC 18:1(Δ11)_18:2(Δ9, 12) |
| 11 | 786.7 | PC 36:2 | 907.7 | 662, 751 | PC 18:1(Δ8)_18:1(Δ8) |
|  |  |  |  | 676, 765 | PC 18:1(Δ9)_18:1(Δ9) |
|  |  |  |  | 704, 793 | PC 18:1(Δ11)_18:1(Δ11) |
|  |  |  |  | 678, 767; 718, 807 | PC 18:0_18:2(Δ9, 12) |
| 12 | 788.7 | PC 36:1 | 909.7 | 678, 767 | PC 18:0_18:1(Δ9), PC 16:0_20:1(Δ11) |
|  |  |  |  | 706, 795 | PC 18:0_18:1(Δ11) |
| 13 | 808.5 | PC 38:5 | 929.5 | 698, 787; 620, 709; 660, 749; 700, 789; 740, 829 | PC 18:1(Δ9)_20:4(Δ5, 8, 11, 14) |
|  |  |  |  | 726, 815; 620, 709; 660, 749; 700, 789; 740, 829 | PC 18:1(Δ11)_20:4(Δ5, 8, 11, 14) |
|  |  |  |  | 622, 711; 662, 751; 702, 791; 742, 831; 782, 871 | PC 18:0_20:5(Δ5, 8, 11, 14, 17), PC 16:0_22:5(Δ7, 10, 13, 16, 19) |
| 14 | 814.8 | PC 38:2 | 935.8 | 704, 793 | PC 18:1(Δ9)_20:1(Δ11) |
|  |  |  |  | 732, 821; 704, 793 | PC 18:1(Δ11)_20:1(Δ11) |


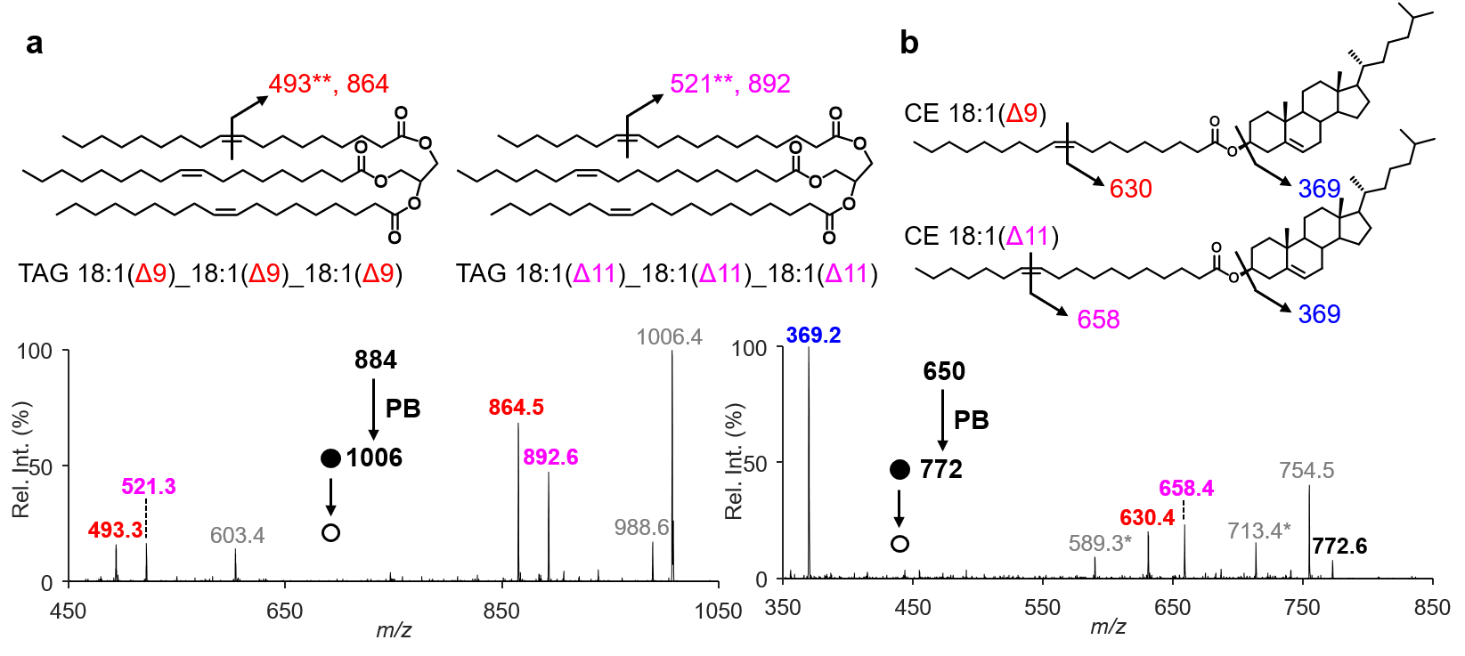


**Supplementary Fig. 11. Tandem MS analysis of derivatized lipids of different classes from a single MCF-7 cell. (a)** TAG 18:1_18:1_18:1 and **(b)** CE 18:1. *Fragment ions characteristic to PCs (peaks with a neutral loss of 59 Da or 183 Da) are detected alongside with CE. This can be explained by the co-isolation of isobaric PC(s) to the lipid of interests. **The diagnostic ions containing aldehyde observed for TAGs were generated by a concomitant loss of water or a fatty acyl after C=C cleavage. All diagnostic ions in the MS/MS spectrum are labeled with the same color as in the corresponding chemical structures.


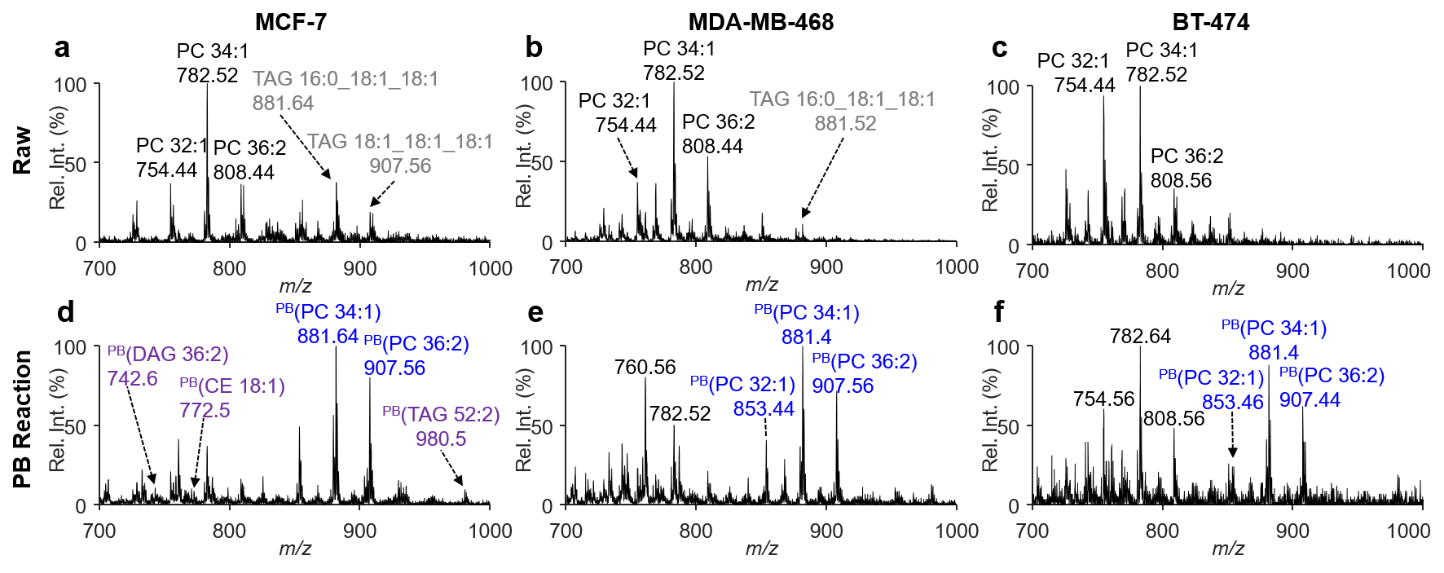


**Supplementary Fig. 12. MS spectrum of a single MCF-7, MDA-MB-468 or BT-474 cell**. **(a)** Mass spectrum of a single intact MCF-7 cell, **(b)** Mass spectrum of a single intact MDA-MB-231 cell, **(c)** Mass spectrum of a single intact BT-474 cell, **(d)** Mass spectrum of a single MCF-7 cell after lipid derivatization, **(e)** Mass spectrum of a single MDA-MB-468 cell after lipid derivatization, **(f)** Mass spectrum of a single BT-474 cell after lipid derivatization. The PB product ions are labeled with blue or purse for PCs or other neutral lipids, respectively.


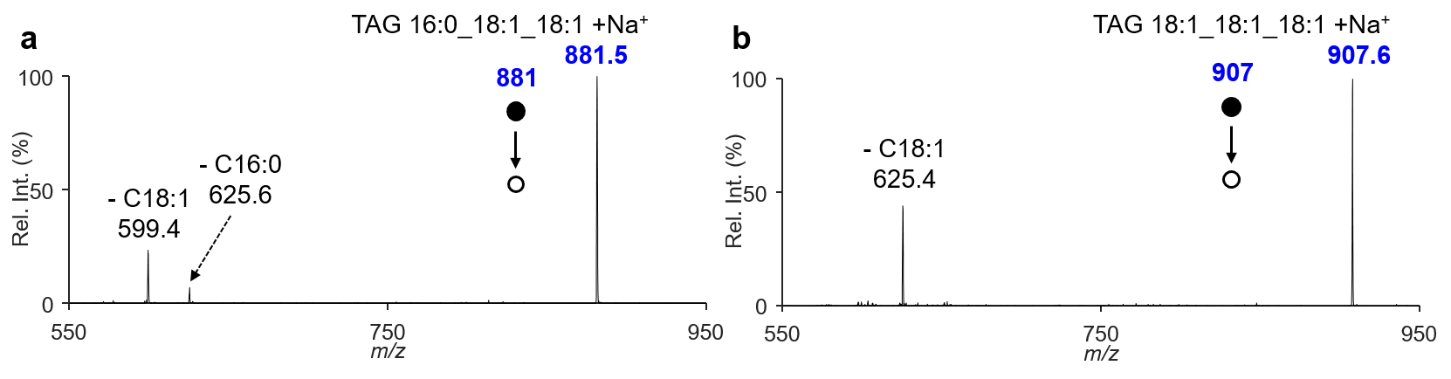


**Supplementary Fig. 13. MS/MS spectra of [TAGs+Na]^+^ from a single MCF-7 cell. (a)** [TAG 16:0_18:1_18:1+ Na]^+^ (*m/z* 881), **(b)** [TAG 18:1_18:1_18:1+ Na]^+^ (*m/z* 907). The precursor ions were labeled with blue.


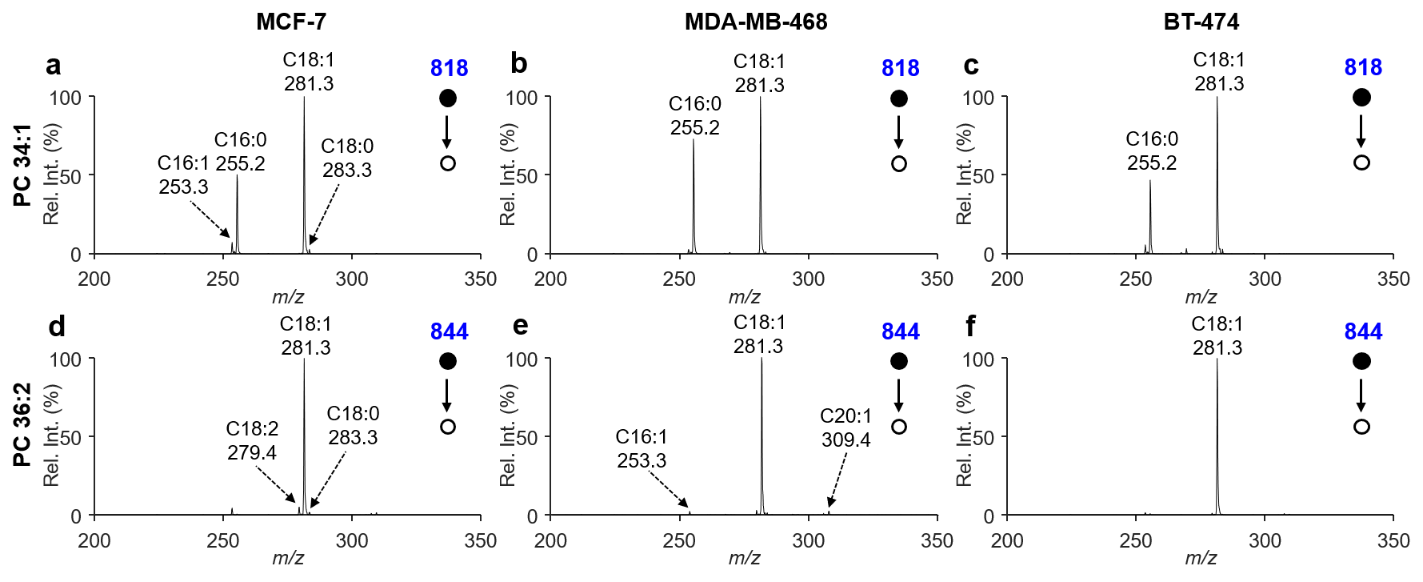


**Supplementary Fig. 14. Negative MS/MS spectra of intact lipids from in MCF-7, MDA-MB-468 and BT-474 cell extracts.** [PC 34:1+CH_3_COO]^-^ (*m/z* 818) from MCF-7 **(a)**, MDA-MB-468 **(b)** and BT-474 **(c)** cells;

[PC 36:2+CH_3_COO]^-^ (*m/z* 844) from MCF-7 **(d)**, MDA-MB-468 **(e)** and BT-474 **(f)** cells.


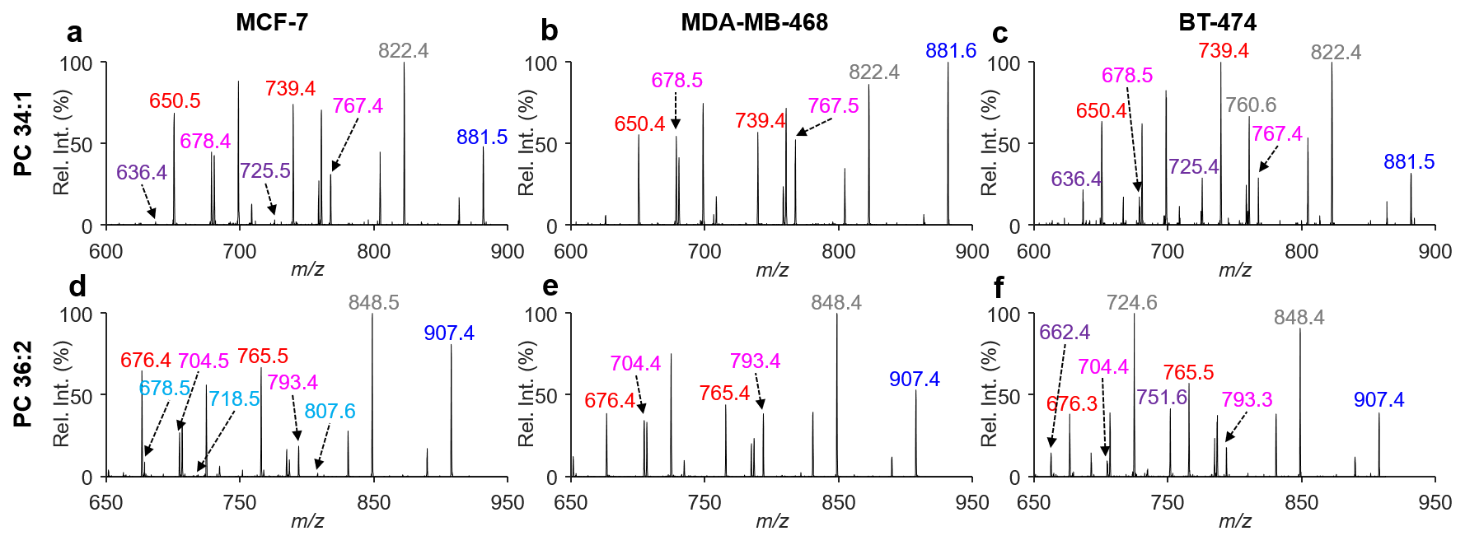


**Supplementary Fig. 15. MS/MS spectra of photochemical products of PCs from MCF-7, MDA-MB-468 and BT-474 single cell.**

^PB^(PC 34:1)(*m/z* 881) from MCF-7 **(a)**, MDA-MB-468 **(b)** and BT-474 **(c)** single cell;

^PB^(PC 36:2)(*m/z* 907) from MCF-7 **(d)**, MDA-MB-468 **(e)** and BT-474 **(f)** single cell.

All the diagnostic ions are marked with colors and are list in Table S2 with isomer assignment.

**Supplementary Table 3.** Fatty acyl chain of lipids in MCF-7 single cells.

| N.O. | m/z | Species | Adduct | Diagnostic Ions | Chain |
| --- | --- | --- | --- | --- | --- |
| 1 | 704.5 | PC 30:1 | 762.5 | 227, 253 | PC 14:0_16:1 |
|  |  |  |  | 225, 255 | PC 14:1_16:0 |
| 2 | 730.5 | PC 32:2 | 788.5 | 253, 253 | PC 16:1_16:1 |
|  |  |  |  | 227, 279 | PC 14:0_18:2 |
|  |  |  |  | 225, 281 | PC 14:1_18:1 |
| 3 | 732.5 | PC 32:1 | 790.5 | 255, 253 | PC 16:0_16:1 |
|  |  |  |  | 227, 281 | PC 14:0_18:1 |
| 4 | 758.5 | PC 34:2 | 816.5 | 253, 281 | PC 16:1_18:1 |
|  |  |  |  | 255, 279 | PC16:0_18:2 |
| 5 | 760.5 | PC 34:1 | 818.5 | 255, 281 | PC 16:0_18:1 |
|  |  |  |  | 253, 283 | PC 16:1_18:0 |
| 6 | 782.5 | PC 36:4 | 840.5 | 255, 303 | PC 16:0_20:4 |
|  |  |  |  | 279, 279 | PC 18:2_18:2 |
| 7 | 784.5 | PC 36:3 | 842.5 | 281, 279 | PC 18:1_18:2 |
|  |  |  |  | 255, 305 | PC 16:0_20:3 |
| 8 | 786.5 | PC 36:2 | 844.5 | 281, 281 | PC 18:1_18:1 |
|  |  |  |  | 253, 309 | PC 16:1_20:1 |
|  |  |  |  | 283, 279 | PC 18:0_18:2 |
| 9 | 788.5 | PC 36:1 | 846.5 | 283, 281 | PC 18:0_18:1 |
|  |  |  |  | 255, 309 | PC 16:0_20:1 |
| 10 | 808.5 | PC 38:5 | 866.5 | 281, 303 | PC 18:1_20:4 |
|  |  |  |  | 255, 329 | PC 16:0_22:5 |
|  |  |  |  | 283, 301 | PC 18:0_20:5 |
| 11 | 810.5 | PC 38:4 | 868.5 | 281, 305 | PC 18:1_20:3 |
|  |  |  |  | 283, 303 | PC 18:0_20:4 |
|  |  |  |  | 255, 331 | PC 16:0_22:4 |
| 12 | 814.5 | PC 38:2 | 872.5 | 281, 309 | PC 18:1_20:1 |

**Supplementary Table 4.** Lipids isomers with C=C locations in single MCF-7 cells.

| N.O. | m/z | Species | PB product | Diagnostic Ions | C=C location |
| --- | --- | --- | --- | --- | --- |
| 1 | 704.5 | PC 30:1 | 825.5 | 594, 683 | PC 14:0_16:1(Δ7) |
|  |  |  |  | 622, 711 | PC 14:0_16:1(Δ9) |
|  |  |  |  | 650, 739 | PC 14:1(Δ9)_16:0 |
| 2 | 730.5 | PC 32:2 | 851.5 | 622, 711; 662, 751 | PC 14:0_18:2(Δ9, 12) |
|  |  |  |  | 676, 765 | PC 14:1(Δ9)_18:1 |
|  |  |  |  | 606, 695 | PC 16:1(Δ6)_16:1(Δ6), PC 14:1_18:1(Δ8) |
|  |  |  |  | 620, 709 | PC 16:1(Δ7)_16:1(Δ7), PC 14:1_18:1(Δ9) |
|  |  |  |  | 648, 737 | PC 16:1(Δ9)_16:1(Δ9), PC 14:1_18:1(Δ11) |
| 3 | 732.5 | PC 32:1 | 853.5 | 608, 697 | PC 14:0_18:1(Δ8), PC 16:0_16:1(Δ6) |
|  |  |  |  | 622, 711 | PC 14:0_18:1(Δ9), PC 16:0_16:1(Δ7) |
|  |  |  |  | 650, 739 | PC 14:0_18:1(Δ11), PC 16:0_16:1(Δ9) |
| 4 | 758.5 | PC 34:2 | 879.5 | 650, 739; 690, 779 | PC 16:0_18:2(Δ9, 12) |
|  |  |  |  | 676, 765 | PC 16:1(Δ9)_18:1(Δ11) |
|  |  |  |  | 648, 737 | PC 16:1(Δ7)_18:1(Δ9) |
| 5 | 760.5 | PC 34:1 | 881.5 | 636, 725 | PC 16:0_18:1(Δ8), PC 16:1(Δ6)_18:0 |
|  |  |  |  | 650, 739 | PC 16:0_18:1(Δ9), PC 16:1(Δ7)_18:0 |
|  |  |  |  | 678, 767 | PC 16:0_18:1(Δ11), PC 16:1(Δ9)_18:0 |
| 6 | 782.5 | PC 36:4 | 903.5 | 594, 683; 634, 723; 674, 763; 714, 803 | PC 16:0_20:4(Δ5, 8, 11, 14) |
|  |  |  |  | 674, 763; 714, 803 | PC 18:2(Δ9, 12)_18:2(Δ9, 12) |
| 7 | 784.5 | PC 36:3 | 905.5 | 636, 725; 676, 765; 716, 805 | PC 16:0_20:3(Δ8, 11, 14) |
|  |  |  |  | 674, 763; 676, 765; 716, 805 | PC 18:1(Δ9)_18:2(Δ9, 12) |
|  |  |  |  | 702, 791; 676, 765; 716, 805 | PC 18:1(Δ11)_18:2(Δ9, 12) |
| 8 | 786.5 | PC 36:2 | 907.5 | 676, 765 | PC 18:1_18:1(Δ9), PC 16:1(Δ7)_20:1(Δ11) |
|  |  |  |  | 704, 793 | PC 18:1_18:1(Δ11), PC 16:1(Δ9)_20:1 |
|  |  |  |  | 678, 767; 718, 807 | PC 18:0_18:2(Δ9, 12) |
| 9 | 788.5 | PC 36:1 | 909.5 | 678, 767 | PC 18:0_18:1(Δ9), PC 16:0_20:1(Δ11) |
|  |  |  |  | 706, 795 | PC 18:0_18:1(Δ11) |
| 10 | 808.5 | PC 38:5 | 929.5 | 698, 787; 620, 709; 660, 749; 700, 789; 740, 829 | PC 18:1(Δ9)_20:4(Δ5, 8, 11, 14) |
|  |  |  |  | 726, 815; 620, 709; 660, 686; 700, 726; 740, 766 | PC 18:1(Δ11)_20:4(Δ5, 8, 11, 14) |
|  |  |  |  | 622, 711; 662, 751; 702, 791; 742, 831; 782, 871 | PC 18:0_20:5(Δ5, 8, 11, 14, 17), PC 16:0_22:5(Δ7, 10, 13, 16, 19) |
| 11 | 810.5 | PC 38:4 | 931.5 | 700, 789; 662, 751; 702, 791; 742, 831 | PC 18:1(Δ9)_20:3(8, 11, 14) |
|  |  |  |  | 728, 817; 662, 751; 702, 791; 742, 831 | PC 18:1(Δ11)_20:3(8, 11, 14) |
|  |  |  |  | 622, 711; 662, 751; 702, 791; 742, 831 | PC 16:0_22:4(Δ7, 10, 13, 16), PC 18:0_20:4(Δ5, 8, 11, 14) |
| 12 | 814.5 | PC 38:2 | 935.5 | 704, 793 | PC 18:1(Δ9)_20:1(Δ11) |
|  |  |  |  | 732, 758 | PC 18:1(Δ11)_20:1 |

**Supplementary Table 5.** Fatty acyl chain of lipids in MDA-MB-468 single cells.

| N.O. | m/z | Species | Adduct | Diagnostic Ions | Chain |
| --- | --- | --- | --- | --- | --- |
| 1 | 732.5 | PC 32:1 | 790.5 | 227, 281 | PC 14:0_18:1 |
|  |  |  |  | 255, 253 | PC 16:0_16:1 |
|  |  |  |  | 241, 267 | PC 15:0_17:1 |
| 2 | 746.5 | PC 33:1 | 804.5 | 255, 267 | PC 16:0_17:1 |
|  |  |  |  | 241, 281 | PC 15:0_18:1 |
|  |  |  |  | 253, 269 | PC 16:1_17:0 |
| 3 | 758.5 | PC 34:2 | 816.5 | 253, 281 | PC 16:1_18:1 |
|  |  |  |  | 255, 279 | PC 16:0_18:2 |
|  |  |  |  | 267, 267 | PC 17:1_17:1 |
| 4 | 760.5 | PC 34:1 | 818.5 | 255, 281 | PC 16:0_18:1 |
|  |  |  |  | 253, 283 | PC 16:1_18:0 |
| 5 | 772.5 | PC 35:2 | 830.5 | 267, 281 | PC 17:1_18:1 |
|  |  |  |  | 269,279 | PC 17:0_18:2 |
| 6 | 774.5 | PC 35:1 | 832.5 | 269, 281 | PC 17:0_18:1 |
|  |  |  |  | 267, 283 | PC 17:1_18:0 |
|  |  |  |  | 241, 309 | PC 15:0_20:1 |
| 7 | 784.5 | PC 36:3 | 842.5 | 281, 279 | PC 18:1_18:2 |
|  |  |  |  | 255, 305 | PC 16:0_20:3 |
|  |  |  |  | 253, 307 | PC 16:1_20:2 |
| 8 | 786.5 | PC 36:2 | 844.5 | 281, 281 | PC 18:1_18:1 |
|  |  |  |  | 253, 309 | PC 16:1_20_1 |
| 9 | 788.5 | PC 36:1 | 846.5 | 283, 281 | PC 18:0_18:1 |
|  |  |  |  | 255, 309 | PC 16:0_20:1 |
| 10 | 810.5 | PC 38:4 | 868.5 | 281, 305 | PC 18:1_20:3 |
|  |  |  |  | 255, 331 | PC 16:0_22:4 |
|  |  |  |  | 283, 303 | PC 18:0_20:4 |
| 11 | 814.5 | PC 38:2 | 872.5 | 281, 309 | PC 18:1_20:1 |

**Supplementary Table 6.** Lipids isomers with C=C locations in single MDA-MB-468 cells.

| N.O. | m/z | Species | PB product | Diagnostic Ions | C=C location |
| --- | --- | --- | --- | --- | --- |
| 1 | 732.5 | PC 32:1 | 853 | 608, 697 | PC 16:0_16:1(Δ6), PC 14:0_18:1(Δ8) |
|  |  |  |  | 622, 711 | PC 16:0_16:1(Δ7), PC 14:0_18:1(Δ9) |
|  |  |  |  | 650, 739 | PC 16:0_16:1(Δ9), PC 14:0_18:1(Δ11) |
|  |  |  |  | 636, 725 | PC 15:0_17:1(Δ9) |
| 2 | 746.5 | PC 33:1 | 867.5 | 622, 711 | PC 15:0_18:1(Δ8), PC 16:1(Δ6)_17:0 |
|  |  |  |  | 636, 725 | PC 15:0_18:1(Δ9), PC 16:1(Δ7)_17:0 |
|  |  |  |  | 664, 753 | PC 15:0_18:1(Δ11), PC 16:1(Δ9)_17:0 |
|  |  |  |  | 650, 739 | PC 16:0_17:1(Δ9) |
| 3 | 758.5 | PC 34:2 | 879.5 | 634, 723 | PC 16:1(Δ6)_18:1(Δ8) |
|  |  |  |  | 648, 737 | PC 16:1(Δ7)_18:1(Δ9) |
|  |  |  |  | 676, 765 | PC 16:1(Δ9)_18:1(Δ11) |
|  |  |  |  | 650, 739; 690, 779 | PC 16:0_18:2(Δ9, 12) |
|  |  |  |  | 662, 751 | PC 17:1(Δ9)_17:1(Δ9) |
| 4 | 760.5 | PC 34:1 | 881.5 | 636, 725 | PC 16:0_18:1(Δ8), PC 16:1(Δ6)_18:0 |
|  |  |  |  | 650, 739 | PC 16:0_18:1(Δ9), PC 16:1(Δ7)_18:0 |
|  |  |  |  | 678, 767 | PC 16:0_18:1(Δ11), PC 16:1(Δ9)_18:0 |
|  |  |  |  | 622, 711 | PC 16:1(Δ6)_17:0 |
| 5 | 772.5 | PC 35:2 | 893.5 | 676, 765 | PC 17:1(Δ9)_18:1 |
|  |  |  |  | 662, 751 | PC 17:1_18:1(Δ9) |
|  |  |  |  | 690, 779 | PC 17:1_18:1(Δ11) |
|  |  |  |  | 664, 753; 704, 793 | PC 17:0_18:2(Δ9, 12) |
| 6 | 774.5 | PC 35:1 | 895.5 | 664, 753 | PC 17:0_18:1(Δ9), PC 15:0_20:1(Δ11) |
|  |  |  |  | 692, 781 | PC 17:0_18:1(Δ11) |
|  |  |  |  | 678, 767 | PC 17:1(Δ9)_18:0 |
| 7 | 784.5 | PC 36:3 | 905.5 | 636, 725; 676, 765; 716, 805 | PC 16:0_20:3(Δ8, 11, 14) |
|  |  |  |  | 674, 763; 676, 765; 716, 805 | PC 18:1(Δ9)_18:2(Δ9, 12), PC 16:1(Δ7)_20:2(Δ11, 14) |
|  |  |  |  | 702, 791; 676, 765; 716, 805 | PC 18:1(Δ11)_18:2(Δ9, 12), PC 16:1(Δ9)_20:2(Δ11, 14) |
| 8 | 786.5 | PC 36:2 | 907.5 | 662, 751 | PC 18:1(Δ8)_18:1(Δ8) |
|  |  |  |  | 676, 765 | PC 18:1_18:1(Δ9), PC 16:1(Δ7)_20:1(Δ11) |
|  |  |  |  | 704, 793 | PC 18:1_18:1(Δ11), PC 16:1(Δ9)_20:1 |
| 9 | 788.5 | PC 36:1 | 909.5 | 678, 767 | PC 18:0_18:1(Δ9), PC 16:0_20:1(Δ11) |
|  |  |  |  | 706, 795 | PC 18:0_18:1(Δ11) |
| 10 | 810.5 | PC 38:4 | 931.5 | 700, 789; 662, 751; 702, 791; 742, 831 | PC 18:1(Δ9)_20:3(8, 11, 14) |
|  |  |  |  | 728, 817; 662, 751; 702, 791; 742, 831 | PC 18:1(Δ11)_20:3(8, 11, 14) |
|  |  |  |  | 622, 711; 662, 751; 702, 791; 742, 831 | PC 16:0_22:4(Δ7, 10, 13, 16), PC 18:0_20:4(Δ5, 8, 11, 14) |
| 11 | 814.8 | PC 38:2 | 935.8 | 704, 793 | PC 18:1(Δ9)_20:1(Δ11) |
|  |  |  |  | 732, 758; 704, 793 | PC 18:1(Δ11)_20:1(Δ11) |

**Supplementary Table 7.** Fatty acyl chain of lipids in BT-474 single cells.

| N.O. | m/z | Species | Adduct | Diagnostic Ions | Chain |
| --- | --- | --- | --- | --- | --- |
| 1 | 704.5 | PC 30:1 | 762.5 | 227, 253 | PC 14:0_16:1 |
| 2 | 730.5 | PC 32:2 | 788.5 | 253, 253 | PC 16:1_16:1 |
| 3 | 732.5 | PC 32:1 | 790.5 | 255, 253 | PC 16:0_16:1 |
|  |  |  |  | 227, 281 | PC 14:0_18:1 |
|  |  |  |  | 241, 267 | PC 15:0_17:1 |
| 4 | 746.5 | PC 33:1 | 804.5 | 255, 267 | PC 16:0_17:1 |
|  |  |  |  | 241, 281 | PC 15:0_18:1 |
|  |  |  |  | 253, 269 | PC 16:1_17:0 |
| 5 | 758.5 | PC 34:2 | 816.5 | 253, 281 | PC 16:1_18:1 |
|  |  |  |  | 255, 279 | PC16:0_18:2 |
|  |  |  |  | 267, 267 | PC 17:1_17:1 |
| 6 | 760.5 | PC 34:1 | 818.5 | 255, 281 | PC 16:0_18:1 |
|  |  |  |  | 253, 283 | PC 16:1_18:0 |
| 7 | 772.5 | PC 35:2 | 830.5 | 267, 281 | PC 17:1_18:1 |
|  |  |  |  | 253, 295 | PC 16:1_19:1 |
| 8 | 774.5 | PC 35:1 | 832.5 | 269, 281 | PC 17:0_18:1 |
|  |  |  |  | 255, 295 | PC 16:0_19:1 |
|  |  |  |  | 253, 297 | PC 16:1_19:0 |
|  |  |  |  | 267, 283 | PC 17:1_18:0 |
| 9 | 784.5 | PC 36:3 | 842.5 | 281, 279 | PC 18:1_18:2 |
|  |  |  |  | 255, 305 | PC 16:0_20:3 |
|  |  |  |  | 253, 307 | PC 16:1_20:2 |
| 10 | 786.5 | PC 36:2 | 844.5 | 281, 281 | PC 18:1_18:1 |
| 11 | 788.5 | PC 36:1 | 846.5 | 283, 281 | PC 18:0_18:1 |
|  |  |  |  | 255, 309 | PC 16:0_20:1 |

**Supplementary Table 8.** Lipids isomers with C=C locations in single BT-474 cells.

| N.O. | m/z | Species | PB product | Diagnostic Ions | C=C location |
| --- | --- | --- | --- | --- | --- |
| 1 | 704.5 | PC 30:1 | 825.5 | 580, 669 | PC 14:0_16:1(Δ6) |
|  |  |  |  | 594, 683 | PC 14:0_16:1(Δ7) |
|  |  |  |  | 622, 711 | PC 14:0_16:1(Δ9) |
| 2 | 730.5 | PC 32:2 | 851.5 | 606, 695 | PC 16:1(Δ6)_16:1(Δ6) |
|  |  |  |  | 620, 709 | PC 16:1(Δ7)_16:1(Δ7) |
|  |  |  |  | 648, 737 | PC 16:1(Δ9)_16:1(Δ9) |
| 3 | 732.5 | PC 32:1 | 853.5 | 608, 697 | PC 16:0_16:1(Δ6) |
|  |  |  |  | 622, 711 | PC 16:0_16:1(Δ7) |
|  |  |  |  | 650, 739 | PC 16:0_16:1(Δ9) |
|  |  |  |  | 636, 725 | PC 15:0_17:1(Δ9) |
| 4 | 746.5 | PC 33:1 | 867.5 | 622, 711 | PC 15:0_18:1(Δ8), PC 16:1(Δ6)_17:0 |
|  |  |  |  | 636, 725 | PC 15:0_18:1(Δ9), PC 16:1(Δ7)_17:0 |
|  |  |  |  | 664, 753 | PC 15:0_18:1(Δ11), PC 16:1(Δ9)_17:0 |
|  |  |  |  | 650, 739 | PC 16:0_17:1(Δ9) |
| 5 | 758.5 | PC 34:2 | 879.5 | 634, 723 | PC 16:1(Δ6)_18:1(Δ8) |
|  |  |  |  | 648, 737 | PC 16:1(Δ7)_18:1(Δ9) |
|  |  |  |  | 676, 765 | PC 16:1(Δ9)_18:1(Δ11) |
|  |  |  |  | 650, 739; 690, 779 | PC 16:0_18:2(Δ9, 12) |
|  |  |  |  | 662, 751 | PC 17:1(Δ9)_17:1(Δ9) |
| 6 | 760.5 | PC 34:1 | 881.5 | 636, 725 | PC 16:0_18:1(Δ8), PC 16:1(Δ6)_18:0 |
|  |  |  |  | 650, 739 | PC 16:0_18:1(Δ9), PC 16:1(Δ7)_18:0 |
|  |  |  |  | 678, 767 | PC 16:0_18:1(Δ11), PC 16:1(Δ9)_18:0 |
| 7 | 772.5 | PC 35:2 | 893.5 | 676, 765; 648, 737 | PC 17:1(Δ9)_18:1(Δ8) |
|  |  |  |  | 676, 765; 662, 751 | PC 17:1(Δ9)_18:1(Δ9) |
|  |  |  |  | 676, 765; 690, 779 | PC 17:1(Δ9)_18:1(Δ11) |
|  |  |  |  | 648, 737; 648, 737 | PC 16:1(Δ6)_19:1(Δ9) |
|  |  |  |  | 662, 751; 648, 737 | PC 16:1(Δ7)_19:1(Δ9) |
|  |  |  |  | 690, 779; 648, 737 | PC 16:1(Δ9)_19:1(Δ9) |
| 8 | 774.5 | PC 35:1 | 895.5 | 650, 739 | PC 17:0_18:1(Δ8), PC 16:1(Δ6)_19:0 |
|  |  |  |  | 664, 753 | PC 17:0_18:1(Δ9), PC 16:1(Δ7)_19:0 |
|  |  |  |  | 692, 781 | PC 17:0_18:1(Δ11), PC 16:1(Δ9)_19:0 |
|  |  |  |  | 678, 767 | PC 17:1(Δ9)_18:0 |
| 9 | 784.5 | PC 36:3 | 905.5 | 636, 725; 676, 765; 716, 805 | PC 16:0_20:3(Δ8, 11, 14) |
|  |  |  |  | 660, 749; 676, 765; 716, 805 | PC 18:1(Δ8)_18:2(Δ9, 12), PC 16:1(Δ6)_20:2(Δ11, 14) |
|  |  |  |  | 674, 763; 676, 765; 716, 805 | PC 18:1(Δ9)_18:2(Δ9, 12), PC 16:1(Δ7)_20:2(Δ11, 14) |
|  |  |  |  | 702, 791; 676, 765; 716, 805 | PC 18:1(Δ11)_18:2(Δ9, 12), PC 16:1(Δ9)_20:2(Δ11, 14) |
| 10 | 786.5 | PC 36:2 | 907.5 | 662, 751 | PC 18:1(Δ8)_18:1(Δ8) |
|  |  |  |  | 676, 765 | PC 18:1(Δ9)_18:1(Δ9) |
|  |  |  |  | 704, 793 | PC 18:1(Δ11)_18:1(Δ11) |
| 11 | 788.5 | PC 36:1 | 909.5 | 664, 753 | PC 18:0_18:1(Δ8) |
|  |  |  |  | 678, 767 | PC 18:0_18:1(Δ9), PC 16:0_20:1(Δ11) |
|  |  |  |  | 706, 795 | PC 18:0_18:1(Δ11) |


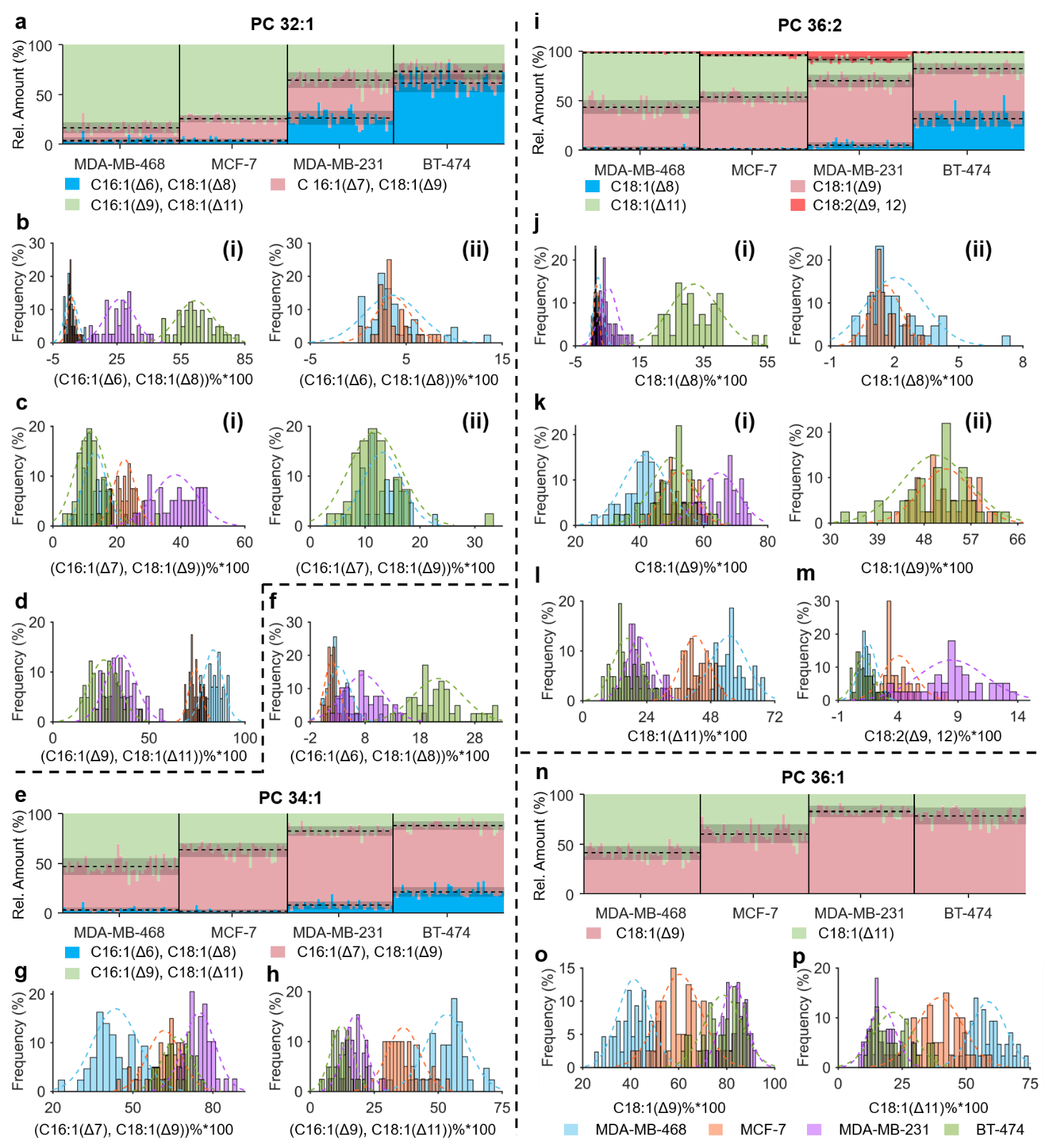


**Supplementary Fig. 16. Relative abundance of structurally defined molecular lipids with C=C location specificity in four different subtypes of human breast cancer cell lines. (a-d)** Overview **(a)** and frequency histogram **(b-d)** of relative abundance of C=C location isomers of PC 32:1 in four types of cells. **(b)** PC 32:1 with C16:1(Δ6) or C18:1(Δ8) in 4 types single cells (i) and MDA-MB-468 and MCF-7 single cells (ii). **(c)** PC 32:1 with C16:1(Δ7) or C18:1(Δ9) in 4 types single cells (i) and MDA-MB-468 and BT-474 single cells (ii). **(d)** PC 32:1 with C16:1(Δ9) or C18:1(Δ11). **(e-h)** Overview **(e)** and frequency histogram **(f-h)** of relative abundance of C=C location isomers of PC 34:1 in four types of cells. **(f)** PC 34:1 with C16:1(Δ6) or C18:1(Δ8). **(g)** PC 34:1 with C16:1(Δ7) or C18:1(Δ9). **(h)** PC 34:1 with C16:1(Δ9) or C18:1(Δ11). **(i-m)** Overview **(i)** and frequency histogram **(j-m)** of relative abundance of C=C location isomers of PC 36:2 in four types of cells. **(j)** PC 36:2 with C18:1(Δ8) in 4 types single cells (i) and MDA-MB-468 and MCF-7 single cells (ii). **(k)** PC 36:2 with C18:1(Δ9) in 4 types single cells (i) and MCF-7 and BT-474 single cells (ii). **(l)** PC 36:2 with C18:1(Δ11). **(m)** PC 18:0_18:2(Δ9, 12). **(n-p)** Overview **(n)** and frequency histogram **(o-p)** of relative abundance of C=C location isomers of PC 36:1 in four types of cells. **(o)** PC 36:1 with C16:1(Δ7) or C18:1(Δ9). **(p)** PC 36:1 with C16:1(Δ9) or C18:1(Δ11). MDA-MB-468: n = 43 single cells, MCF-7: n = 40 single cells, MDA-MB-231: n = 39 single cells, BT-474: n = 41 single cells. Source data are provided in a Source Data file.

**Supplementary Table 9.** The significant difference for the relative amount of PC species between each two breast cancer cell lines. The *p* values (calculated using two-tailed Student’s *t* test) highlighted in red indicate no statistical differences (*p*>0.05). MDA-MB-468: n = 43 single cells, MCF-7: n = 40 single cells, MDA-MB-231: n = 39 single cells, BT-474: n = 41 single cells.

| Lipid species | MDA-MB-468 v.s. MCF-7 | MCF-7 v.s. MDA-MB-231 | MDA-MB-231 v.s. BT-474 | MDA-MB-468 v.s. MDA-MB-231 | MCF-7 v.s. BT-474 | MDA-MB-468 v.s. BT-474 |
| --- | --- | --- | --- | --- | --- | --- |
| PC 16:0_16:1(Δ6) | 0.803909729 | 1.01E-32 | 6.43E-33 | 4.68E-33 | 9.35E-56 | 2.38E-57 |
| PC 16:0_16:1(Δ7) | 5.03E-20 | 1.08E-21 | 9.28E-33 | 1.17E-34 | 1.35E-18 | 0.141323 |
| PC 16:0_16:1(Δ9) | 5.56E-16 | 4.06E-43 | 2.27E-06 | 5.72E-49 | 1.44E-50 | 9.35E-56 |
| PC 16:1(Δ6)_18:1(Δ8) | 0.715623154 | 1.37E-19 | 4.74E-28 | 3.26E-19 | 2.77E-44 | 1.60E-45 |
| PC 16:1(Δ7)_18:1(Δ9) | 4.72E-07 | 1.28E-20 | 9.01E-16 | 2.80E-28 | 0.249806579 | 2.42E-07 |
| PC 16:1(Δ9)_18:1(Δ11) | 3.50E-21 | 3.60E-29 | 0.367967759 | 5.83E-44 | 3.17E-32 | 4.36E-47 |
| PC 16:0_18:2(Δ9, 12) | 3.68E-28 | 0.168965904 | 5.71E-37 | 4.78E-34 | 4.63E-31 | 3.05E-05 |
| PC 16:0_18:1(Δ8), PC 16:1(Δ6)_18:0 | 0.001014083 | 6.46E-14 | 3.52E-24 | 1.80E-09 | 1.49E-40 | 1.07E-38 |
| PC 16:0_18:1(Δ9), PC 16:1(Δ7)_18:0 | 7.55E-18 | 9.24E-15 | 4.75E-09 | 7.88E-32 | 0.000475164 | 1.04E-24 |
| PC 16:0_18:1(Δ11), PC 16:1(Δ9)_18:0 | 1.06E-16 | 4.94E-25 | 6.89E-07 | 7.17E-39 | 1.13E-32 | 1.41E-44 |
| PC 18:1(Δ8)_18:1(Δ8) | 0.063287783 | 3.82E-12 | 2.08E-33 | 1.58E-09 | 1.88E-39 | 1.41E-40 |
| PC 18:1(Δ9)_18:1(Δ9) | 9.78E-12 | 1.24E-15 | 3.50E-16 | 1.13E-26 | 0.130816599 | 9.64E-08 |
| PC 18:1(Δ11)_18:1(Δ11) | 8.16E-15 | 3.76E-29 | 4.04E-05 | 7.71E-40 | 1.10E-35 | 4.28E-45 |
| PC 18:0_18:2(Δ9, 12) | 6.38E-18 | 1.62E-13 | 1.99E-26 | 2.78E-26 | 1.74E-19 | 0.054808678 |
| PC 18:0_18:1(Δ9) | 1.32E-17 | 3.29E-22 | 0.003675384 | 5.49E-46 | 4.72E-15 | 1.03E-37 |
| PC 18:0_18:1(Δ11) | 1.32E-17 | 3.29E-22 | 0.003675384 | 5.49E-46 | 4.72E-15 | 1.03E-37 |


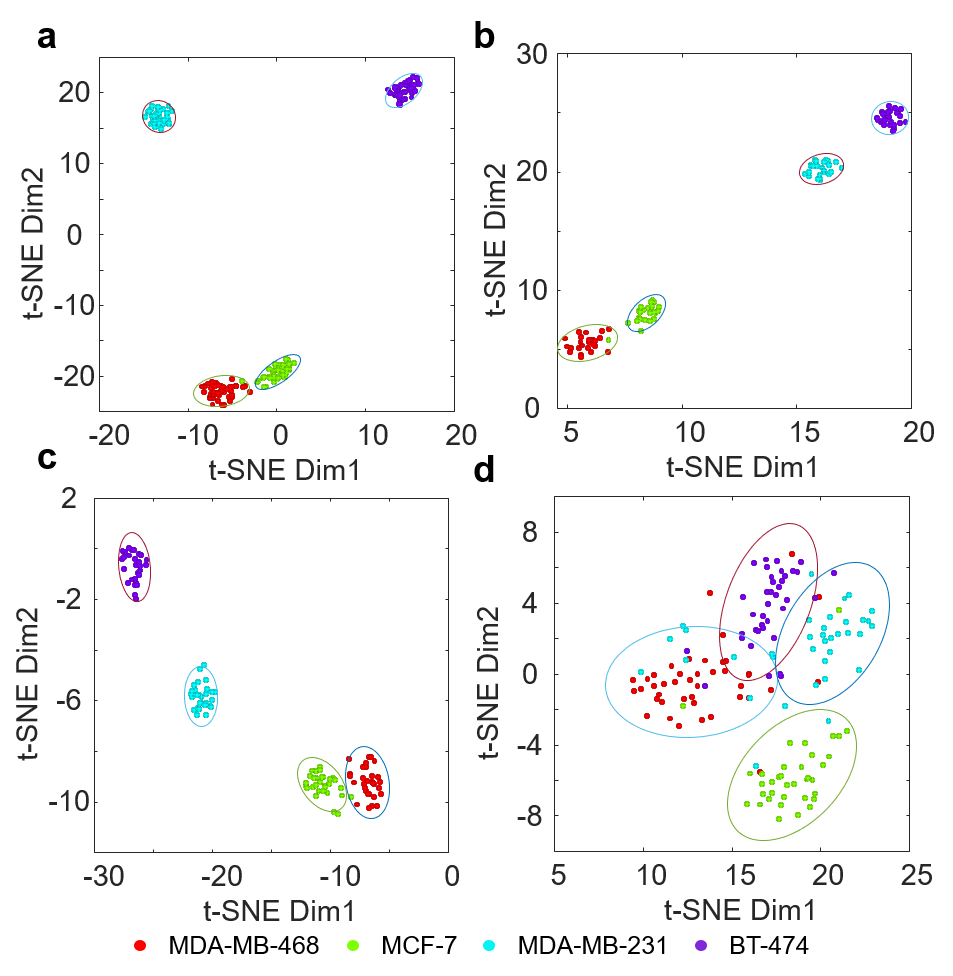


**Supplementary Fig. 17. T-SNE plot of different subtypes of human breast cancer single cells calculated by relative quantitation of lipid C=C location isomers (a-c) or *sn*-position isomers (b).** (b-c) T-SNE plot of 25 (b) or 30 (c) single cells selected randomly from each cell lines. The cluster areas were circled by k-means clustering results with a confidence probability of 0.95. Number of cells: (a) MDA-MB-468: n = 43 single cells, MCF-7: n = 40 single cells, MDA-MB-231: n = 39 single cells, BT-474: n = 41 single cells; (b) MDA-MB-468: n = 25 single cells, MCF-7: n = 25 single cells, MDA-MB-231: n = 25 single cells, BT-474: n = 25 single cells; (c) MDA-MB-468: n = 30 single cells, MCF-7: n = 30 single cells, MDA-MB-231: n = 30 single cells, BT-474: n = 30 single cells; (d) MDA-MB-231: n = 42 single cells, MCF-7: n = 36 single cells, MDA-MB-231: n = 36 single cells, BT-474: n = 38 single cells. Source data are provided in a Source Data file.

**Supplementary Note 1. Response of cellular lipidome to SCD1 inhibition.**

Stearoyl-CoA desaturase (SCD1) is the rate-limiting enzyme in the biosynthesis of monounsaturated fatty acids, e.g. FA 16:1 and FA 18:1 (Supplementary Fig. 18a), and these fatty acids are further incorporated into other classes of lipids such as PC, PE, PS, and PI ^1^. The overall increase in lipid desaturation observed in a variety of cancers have stimulated increased research interests in developing SCD1 inhibitors for cancer treatment. However, the effect of SCD1 inhibition on the lipid composition in mammalian cells at detailed structure level remains unknown.

In our study, MDA-MB-468 cells were treated with an SCD1 inhibitor, i.e. CAY10566, for probing the relative changes in C=C location isomers of PCs at the single cell level^2^. As expected, the relative amounts of lipid C=C location isomers changed significantly after cultured with 100 nM CAY10566 (Fig. Supplementary Fig. 18b, Supplementary Fig. S19, Table 10). Particularly, the relative amounts of PC 34:2 with C16:1(Δ7) or C18:1(Δ9) were relatively stable (Supplementary Fig. 18c), while those of PC 34:2 with C16:1(Δ9) or C18:1(Δ11) decreased significantly (*P* < 0.001, determined by two-tailed *t*-test) (Supplementary Fig. 18d). Fatty acyl C16:1(Δ7) could not be directly synthesized via desaturation of C16:0 but produced after *β*-oxidation of C18:1(Δ9). Following SCD1 inhibition, the relative amounts of PC 34:2 with C16:1(Δ6) or C18:1(Δ8) also increased (*P* < 0.001, determined by two-tailed *t*-test) (Supplementary Fig. 18e). Both fatty acyls were synthesized by the desaturase FADS2, which was complementary to SCD1/SCD5-involved lipid desaturation pathways in human cells and was reported to be associated with cancer pathology^3^. Since the biosynthesis of monounsaturated fatty acyls was inhibited, the relative amount of C18:2(Δ9, 12) also increased (*P* < 0.001, determined by two-tailed *t*-test) (Supplementary Fig. 18f). Similar results were observed for PC 32:1 and PC 36:2 (Supplementary, Fig. 19). It should be noted that, the relative amounts of PC 34:1 with C18:1(Δ9) increased, which might be due to the decrease of PC 34:1 with C18:1(Δ11) (Supplementary, Fig. 19g-f).


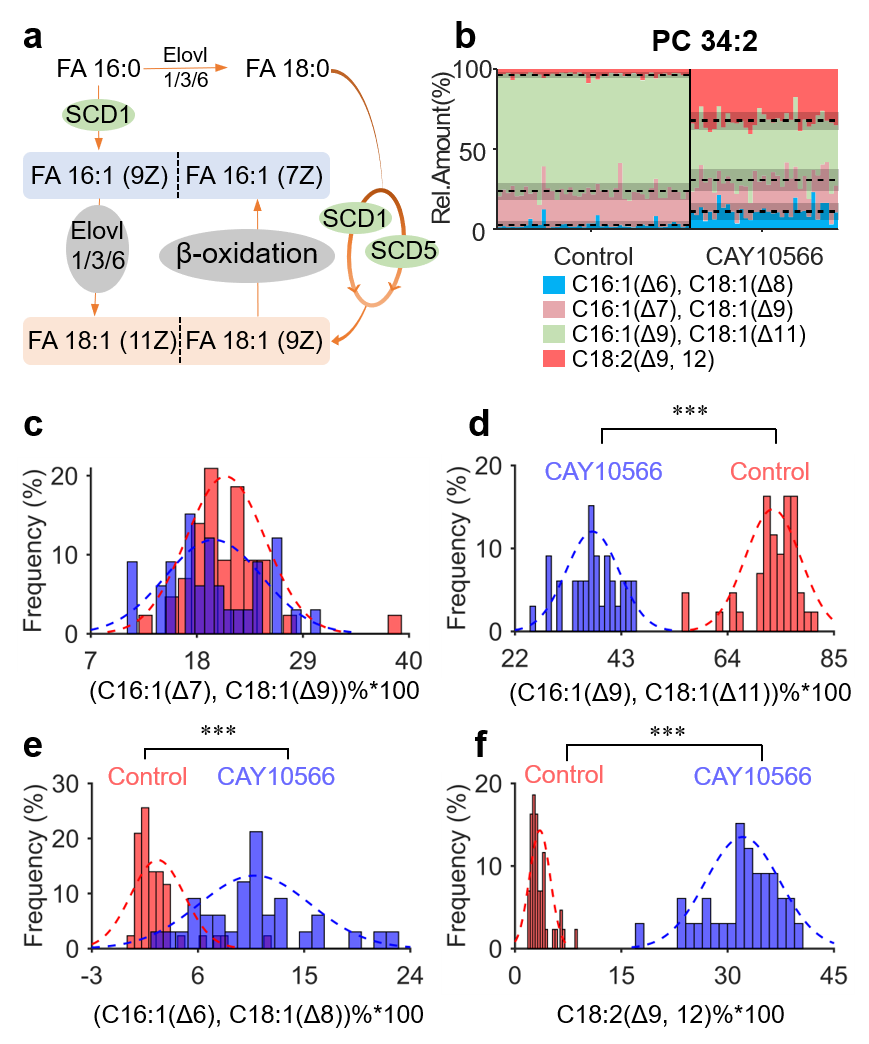


**Supplementary Fig. 18. Changes of the cell lipidome in response to SCD1 inhibition. (a)** Pathway of monounsaturated fatty acids biosynthesis. **(b-f)** Overview **(b)** and frequency histogram **(c-f)** of relative amounts of C=C location isomers of PC 34:2 in single MDA-MB-231 cells after CAY10566 treatment. **(c)** PC 34:2 with C16:1(Δ7) or C18:1(Δ9), **(d)** PC 34:2 with C16:1(Δ9) or C18:1(Δ11), **(e)** PC 34:2 with C16:1(Δ6) or C18:1(Δ8) and **(f)** PC 16:0_18:2(Δ9, 12). *** *P* < 0.001, determined by two-tailed *t*-test. Control: n = 43 single cells, CAY10566: n = 33 single cells. See exact *p*-values in Supplementary Table 10. Source data are provided in a Source Data file.


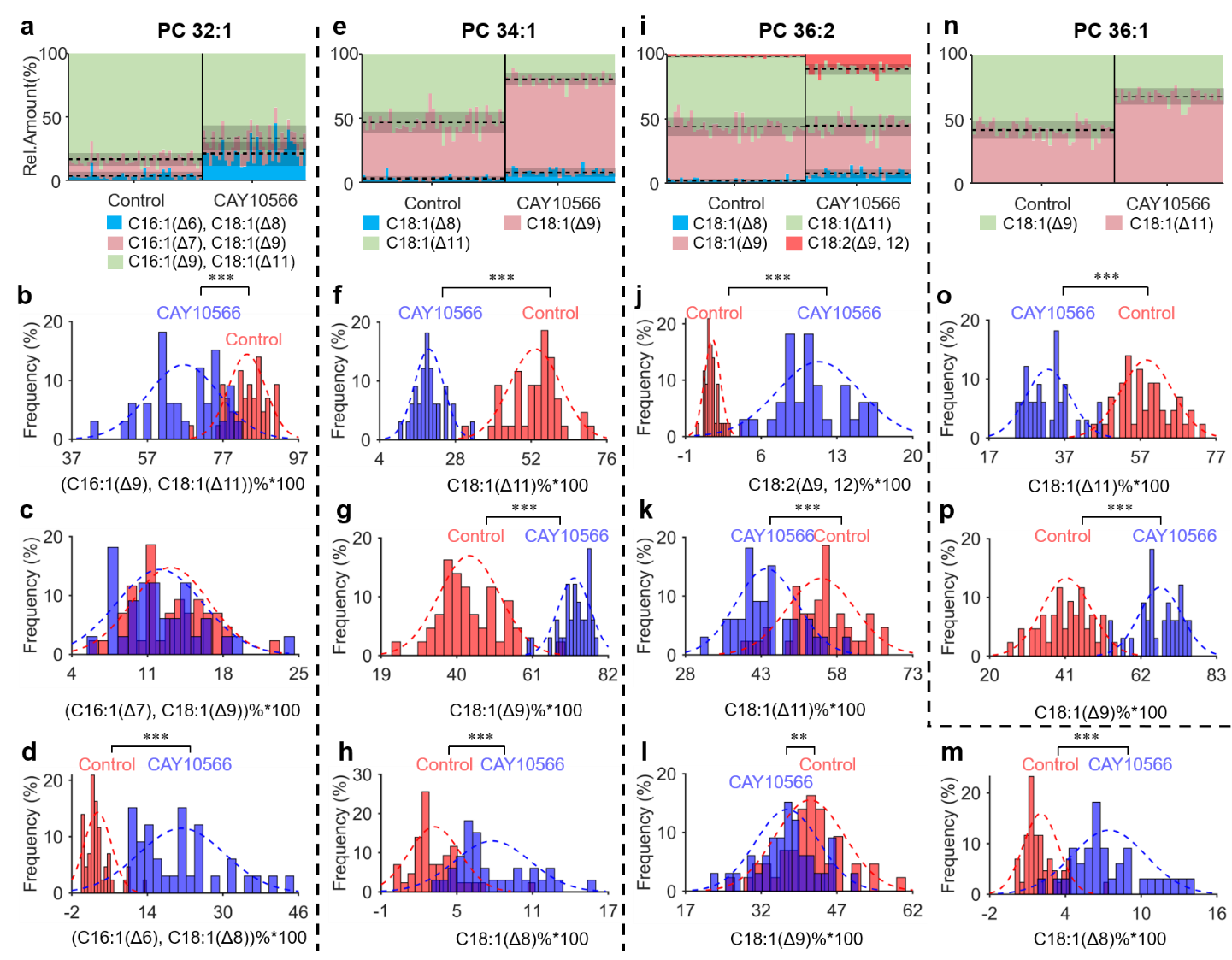


**Supplementary Fig. 19. Alterations in the relative amounts of PC isomers in single MDA-MB-468 cells upon SCD1 inhibition. (a-d)** PC 32:1. **(a)** Overview of the relative amounts of different PC 32:1 isomers, **(b-d)** Histogram of cells according to the relative amounts of PC 32:1 isomers. **(b)** PC 32:1 with C16:1(Δ9) or C18:1(Δ11), **(c)** PC 32:1 with C16:1(Δ7) or C18:1(Δ9) and **(d)** PC 32:1 with C16:1(Δ6) or C18:1(Δ8). **(e-h)** PC 34:1. **(e)** Overview of the relative amounts of different PC 34:1 isomers, **(f-h)** Histogram of cells according to the relative amounts of PC 34:1 isomers. **(f)** PC 34:1 with C18:1(Δ11), **(g)** PC 34:1 with C18:1(Δ9) and **(h)** PC 34:1 with C18:1(Δ8). **(i-m)** PC 36:2. **(i)** Overview of the relative amounts of different PC 36:2 isomers, **(j-m)** Histogram of cells according to the relative amounts of PC 36:2 isomers. **(j)** PC 18:0_ 18:2(Δ9, 12), **(k)** PC 36:2 with C18:1(Δ11), **(l)** PC 36:2 with C18:1(Δ9) and **(m)** PC 36:2 with C18:1(Δ8). **(n-p)** PC 36:1. **(n)** Overview of the relative amounts of different PC 36:1 isomers, **(o-p)** Histogram of cells according to the relative amounts of PC 36:1 isomers. **(o)** PC 36:1 with C18:1(Δ11) and **(p)** PC 36:1 with C18:1(Δ9). ** *p* < 0.01, *** *p* < 0.001, determined by two-tailed *t*-test. Control: n = 43 single cells, CAY10566: n = 33 single cells. See exact *p*-values in Supplementary Table 10. Source data are provided in a Source Data file.

**Supplementary Table 10.** The significant difference for the relative amount of PC species between MDA-MB-468 single cells with and without drug treatment. The *p* values (calculated using two-tailed Student’s *t* test) highlighted in red indicate no statistical differences (*p*>0.05). Control: n = 43 single cells, CAY10566: n = 33 single cells.

| Lipid species | MDA-MB-468. v.s. MDA-MB-468 with drug treatment |
| --- | --- |
| PC 16:0_16:1(Δ6) | 4.33E-19 |
| PC 16:0_16:1(Δ7) | 0.242071142 |
| PC 16:0_16:1(Δ9) | 2.46E-15 |
| PC 16:1(Δ6)_18:1(Δ8) | 2.16E-15 |
| PC 16:1(Δ7)_18:1(Δ9) | 0.240043685 |
| PC 16:1(Δ9)_18:1(Δ11) | 6.79E-42 |
| PC 16:0_18:2(Δ9, 12) | 3.95E-47 |
| PC 16:0_18:1(Δ8)/PC 16:1(Δ6)_18:0 | 1.36E-11 |
| PC 16:0_18:1(Δ9)/PC 16:1(Δ7)_18:0 | 1.36E-28 |
| PC 16:0_18:1(Δ11)/PC 16:1(Δ9)_18:0 | 3.56E-33 |
| PC 18:1(Δ8)_18:1(Δ8) | 1.55E-16 |
| PC 18:1(Δ9)_18:1(Δ9) | 0.004749234 |
| PC 18:1(Δ11)_18:1(Δ11) | 3.93E-10 |
| PC 18:0_18:2(Δ9, 12) | 1.99E-26 |
| PC 18:0_18:1(Δ9) | 5.71E-28 |
| PC 18:0_18:1(Δ11) | 5.71E-28 |

**Supplementary Table 11.** Lipids isomers with *sn*-positions for relative quantitation in single cells.

| N.O. | Molecular lipid | Adduct | Diagnostic Ions | *sn*-position isomers |
| --- | --- | --- | --- | --- |
| 1 | PC 16:0_16:1 | 792.5 | 419 | PC 16:0/16:1 |
|  |  |  | 417 | PC 16:1/16:0 |
| 2 | PC 16:1_18:1 | 818.5 | 417 | PC 16:1/18:1 |
|  |  |  | 445 | PC 18:1/16:1 |
| 3 | PC 16:0_18:2 | 818.5 | 419 | PC 16:0/18:2 |
|  |  |  | 443 | PC 18:2/16:0 |
| 4 | PC 16:0_18:1 | 820.5 | 419 | PC 16:0/18:1 |
|  |  |  | 445 | PC 18:1/16:0 |
| 5 | PC 16:1_18:0 | 820.5 | 417 | PC 16:1/18:0 |
|  |  |  | 447 | PC 18:0/16:1 |
| 6 | PC 18:0_18:1 | 848.5 | 445 | PC 18:0/18:1 |
|  |  |  | 447 | PC 18:1/18:0 |


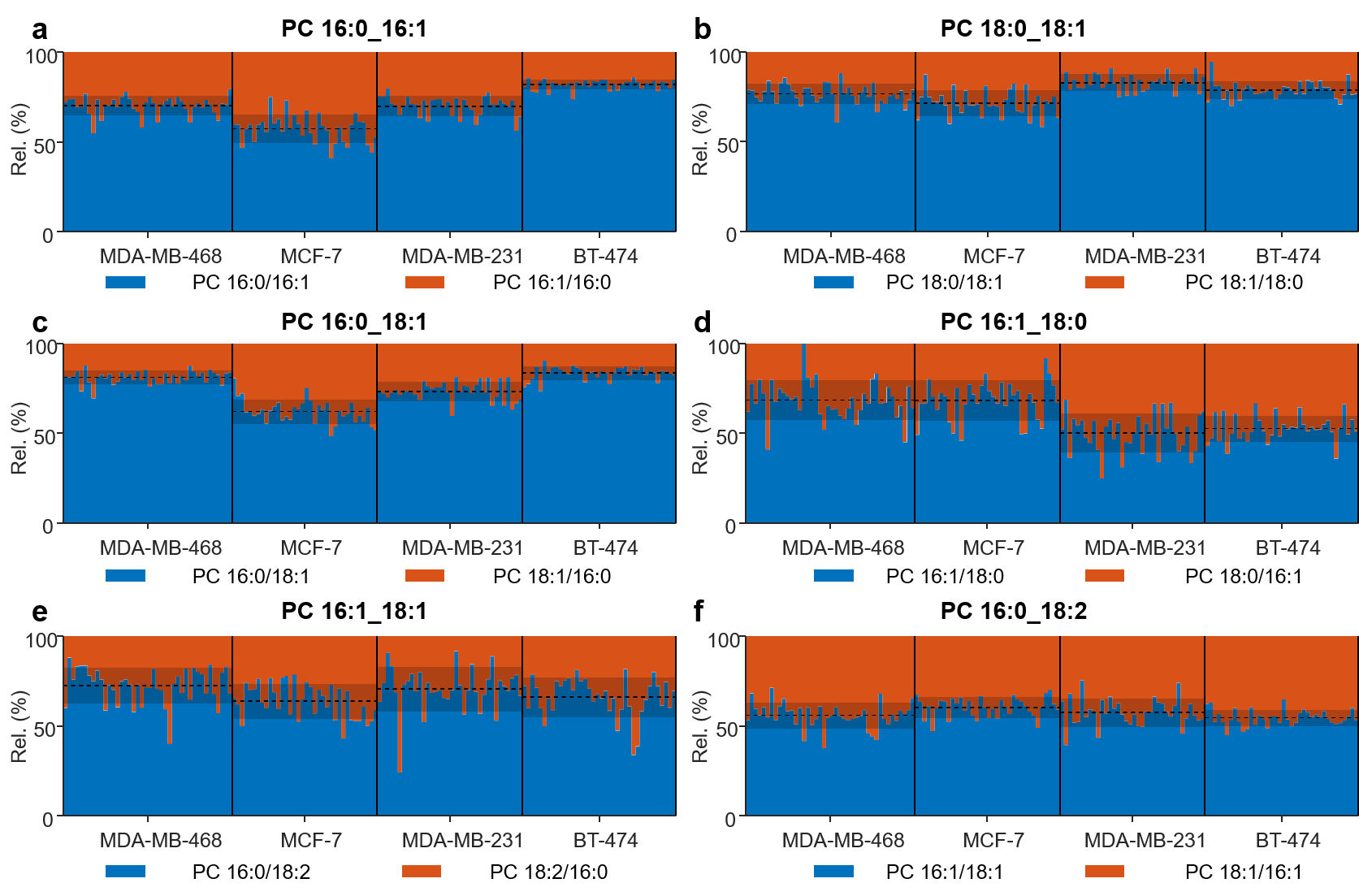


**Supplementary Fig. 20. Relative abundance of pairwise lipid *sn*-position isomers in four different subtypes of human breast cancer cell lines. (a)** PC 16:0_16:1, **(b)** PC 18:0_18:1, **(c)** PC 16:0_18:1, **(d)** PC 16:1_18:0, **(e)** PC 16:1_18:1, **(f)** PC 16:0_18:2. MDA-MB-468: n = 42 single cells, MCF-7: n = 36 single cells, MDA-MB-231: n = 36 single cell, BT-474: n = 38 single cells. Source data are provided in a Source Data file.

**Supplementary Table 12.** The significant difference for the relative amount of PC species with *sn*-positions between each two breast cancer cell lines. The *p* values (calculated using two-tailed Student’s *t* test) highlighted in red indicate no statistical differences (*p*>0.05). MDA-MB-468: n = 42 single cells, MCF-7: n = 36 single cells, MDA-MB-231: n = 36 single cell, BT-474: n = 38 single cells.

| Lipid species | MDA-MB-468 v.s. MCF-7 | MCF-7 v.s. MDA-MB-231 | MDA-MB-231 v.s. BT-474 | MDA-MB-468 v.s. MDA-MB-231 | MCF-7 v.s. BT-474 | MDA-MB-468 v.s. BT-474 |
| --- | --- | --- | --- | --- | --- | --- |
| PC 16:0/16:1 | 4.47e-13 | 9.50e-12 | 1.62E-19 | 0.912457258 | 2.20e-29 | 1.59E-20 |
| PC 16:1/16:0 | 4.47e-13 | 9.50e-12 | 1.62E-19 | 0.912457258 | 2.20e-29 | 1.59E-20 |
| PC 16:1/18:1 | 0.002596022 | 0.08217976 | 0.037442677 | 0.302363514 | 3.59e-06 | 0.314477435 |
| PC 18:1/16:1 | 0.002596022 | 0.08217976 | 0.03744267 | 0.302363514 | 3.59e-06 | 0.314477435 |
| PC 16:0/18:2 | 0.000176828 | 0.00954802 | 0.101347147 | 0.474129947 | 0.3106412 | 0.008400516 |
| PC 18:2/16:0 | 0.000176828 | 0.00954802 | 0.101347147 | 0.474129947 | 0.3106412 | 0.008400516 |
| PC 16:0/18:1 | 7.89e-26 | 2.46e-11 | 3.27E-15 | 3.08E-11 | 2.03e-27 | 0.002833595 |
| PC 18:1/16:0 | 7.89e-26 | 2.46e-11 | 3.27E-15 | 3.08E-11 | 2.03e-27 | 0.002833595 |
| PC 16:1/18:0 | 0.903886237 | 1.04e-09 | 0.232133823 | 8.41E-11 | 3.25e-10 | 2.95E-11 |
| PC 18:0/16:1 | 0.903886237 | 1.04e-09 | 0.232133823 | 8.41E-11 | 3.25e-10 | 2.95E-11 |
| PC 18:0/18:1 | 0.000328699 | 5.86e-12 | 2.57E-04 | 6.18E-07 | 1.05e-06 | 0.072312236 |
| PC 18:1/18:0 | 0.000328699 | 5.86e-12 | 2.57E-04 | 6.18E-07 | 1.05e-06 | 0.072312236 |


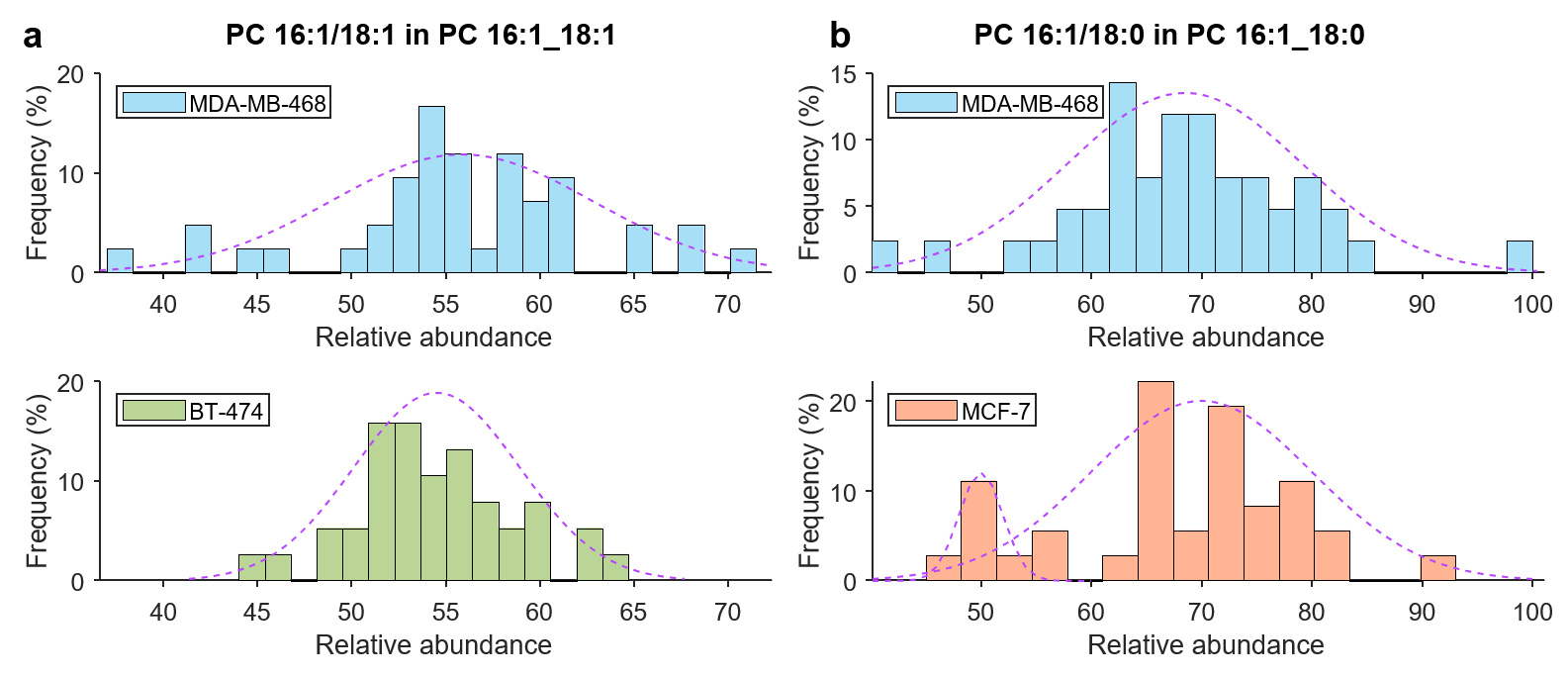


**Supplementary Fig. 21. Histogram of cells according to the relative amounts of PC 16:1/18:1 in PC 16:1_18:1 (a) and PC 16:1/18:0 in PC 16:1_18:0 (b).** MDA-MB-468: n = 42 single cells, MCF-7: n = 36 single cells, BT-474: n = 38 single cells. Source data are provided in a Source Data file.


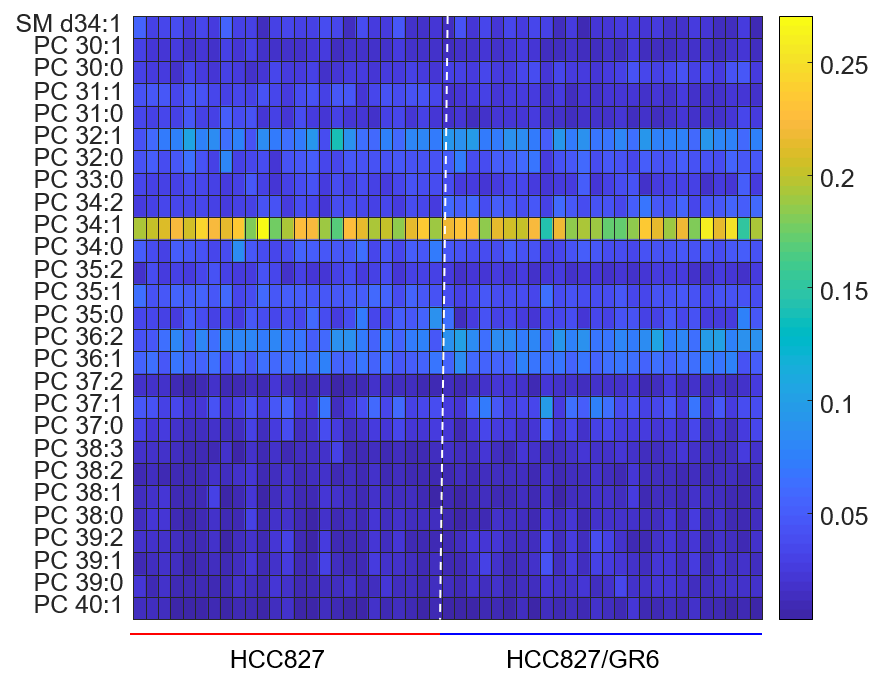


**Supplementary Fig. 22. Heatmap of the relative abundance of lipid sum compositions in HCC827 and HCC827/GR6 single cells.** HCC827: n = 25 single cells, HCC827/GR6: n = 26 single cells. Source data are provided in a Source Data file.

**Supplementary Table 13.** The significant difference for the relative amount of PC species between HCC827 and HCC827/GR6 single cells. The *p* values (calculated using two-tailed Student’s *t* test) highlighted in red indicate no statistical differences (*p*>0.05). HCC827: n = 25 single cells, HCC827/GR6: n = 26 single cells.

| Lipid species | Li^+^ adduct | HCC827 v.s. HCC827/GR6 |
| --- | --- | --- |
| SM d34:1 | 709.5 | 0.00270450 |
| PC 30:1 | 710.5 | 0.00298137 |
| PC 30:0 | 712.5 | 0.00094336 |
| PC 31:1 | 724.5 | 2.12e-14 |
| PC 31:0 | 726.5 | 3.90e-07 |
| PC 32:1 | 738.5 | 0.40093300 |
| PC 32:0 | 740.5 | 0.12659613 |
| PC 33:0 | 754.5 | 0.10756453 |
| PC 34:2 | 764.5 | 5.59e-09 |
| PC 34:1 | 766.5 | 0.34603958 |
| PC 34:0 | 768.5 | 0.11094964 |
| PC 35:2 | 778.5 | 3.70e-05 |
| PC 35:1 | 780.5 | 2.69e-05 |
| PC 35:0 | 782.5 | 0.14524336 |
| PC 36:2 | 792.5 | 0.00034207 |
| PC 36:1 | 794.5 | 0.07383691 |
| PC 37:2 | 806.5 | 0.00017944 |
| PC 37:1 | 808.5 | 0.13494342 |
| PC 37:0 | 810.5 | 0.27572683 |
| PC 38:3 | 818.5 | 0.00578737 |
| PC 38:2 | 820.5 | 0.91030815 |
| PC 38:1 | 822.5 | 0.51712136 |
| PC 38:0 | 824.5 | 0.84955944 |
| PC 39:2 | 834.5 | 0.09464154 |
| PC 39:1 | 836.5 | 0.037739142 |
| PC 39:0 | 838.5 | 0.003772297 |
| PC 40:1 | 850.5 | 0.649088883 |


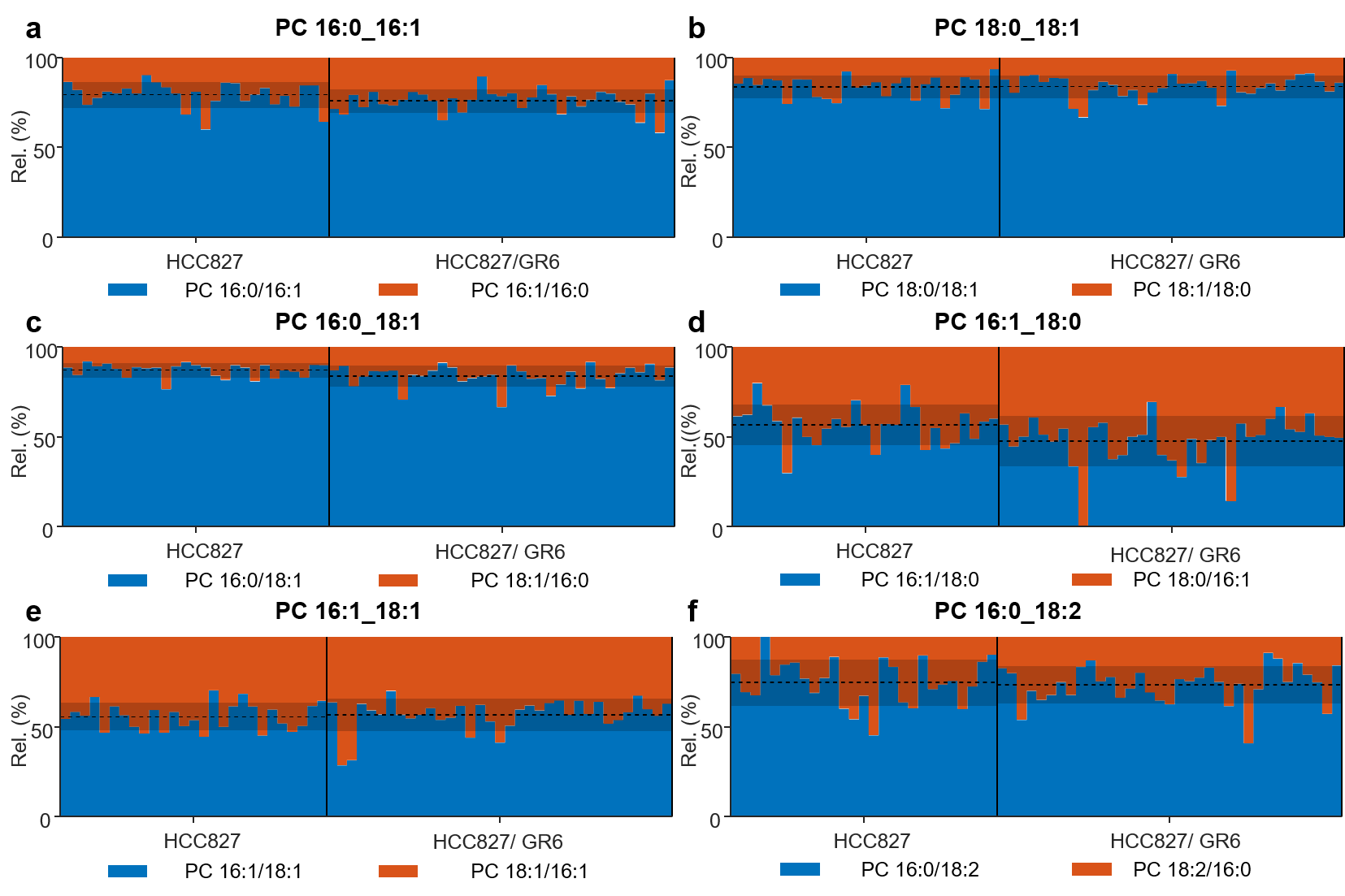


**Supplementary Fig. 23. Relative abundance of pairwise lipid *sn*-position isomers in HCC827 and HCC827/GR6 single cells. (a)** PC 16:0_16:1, **(b)** PC 18:0_18:1, **(c)** PC 16:0_18:1, **(d)** PC 16:1_18:0, **(e)** PC 16:1_18:1, **(f)** PC 16:0_18:2. HCC827: n = 27 single cells, HCC827/GR6: n = 35 single cells. Source data are provided in a Source Data file.

**Supplementary Table 14.** The significant difference for the relative amount of PC species with *sn*-positions between HCC827 and HCC827/GR6 single cells. The *p* values (calculated using two-tailed Student’s *t* test) highlighted in red indicate no statistical differences (*p*>0.05). HCC827: n = 27 single cells, HCC827/GR6: n = 35 single cells.

| Lipid species | HCC827 v.s. HCC827/GR6 |
| --- | --- |
| PC 16:0/16:1 | 0.058898302 |
| PC 16:1/16:0 | 0.058898302 |
| PC 16:1/18:1 | 0.632326438 |
| PC 18:1/16:1 | 0.632326438 |
| PC 16:0/18:2 | 0.644421813 |
| PC 18:2/16:0 | 0.644421813 |
| PC 16:0/18:1 | 0.010519699 |
| PC 18:1/16:0 | 0.010519699 |
| PC 16:1/18:0 | 0.007112698 |
| PC 18:0/16:1 | 0.007112698 |
| PC 18:0/18:1 | 0.879503784 |
| PC 18:1/18:0 | 0.879503784 |

**Supplementary Table 15.** Lipids isomers with C=C locations for relative quantitation in HCC827 and HCC827/GR6 single cells.

| N.O. | m/z | Species | PB product | Diagnostic Ions | C=C location |
| --- | --- | --- | --- | --- | --- |
| 1 | 732.5 | PC 32:1 | 853.5 | 608, 697 | PC 16:0_16:1(Δ6), PC 14:0_18:1(Δ8) |
|  |  |  |  | 622, 711 | PC 16:0_16:1(Δ7) , PC 14:0_18:1(Δ9) |
|  |  |  |  | 650, 739 | PC 16:0_16:1(Δ9) , PC 14:0_18:1(Δ11) |
| 2 | 758.5 | PC 34:2 | 879.5 | 634, 723 | PC 16:1(Δ6)_18:1(Δ8) |
|  |  |  |  | 648, 737 | PC 16:1(Δ7)_18:1(Δ9) |
|  |  |  |  | 676, 765 | PC 16:1(Δ9)_18:1(Δ11) |
|  |  |  |  | 650, 739; 690, 779 | PC 16:0_18:2(Δ9, 12) |
| 3 | 760.5 | PC 34:1 | 881.5 | 636, 725 | PC 16:0_18:1(Δ8), PC 16:1(Δ6)_18:0 |
|  |  |  |  | 650, 739 | PC 16:0_18:1(Δ9), PC 16:1(Δ7)_18:0 |
|  |  |  |  | 678, 767 | PC 16:0_18:1(Δ11), PC 16:1(Δ9)_18:0 |
| 4 | 786.5 | PC 36:2 | 907.5 | 662, 751 | PC 18:1(Δ8)_18:1(Δ8) |
|  |  |  |  | 676, 765 | PC 18:1(Δ9)_18:1(Δ9) |
|  |  |  |  | 704, 793 | PC 18:1(Δ11)_18:1(Δ11) |
|  |  |  |  | 678, 767, 718, 807 | PC 18:0_18:2(Δ9, 12) |
| 5 | 788.5 | PC 36:1 | 909.5 | 664, 753 | PC 18:0_18:1(Δ8) |
|  |  |  |  | 678, 767 | PC 18:0_18:1(Δ9) |
|  |  |  |  | 706, 795 | PC 18:0_18:1(Δ11) |


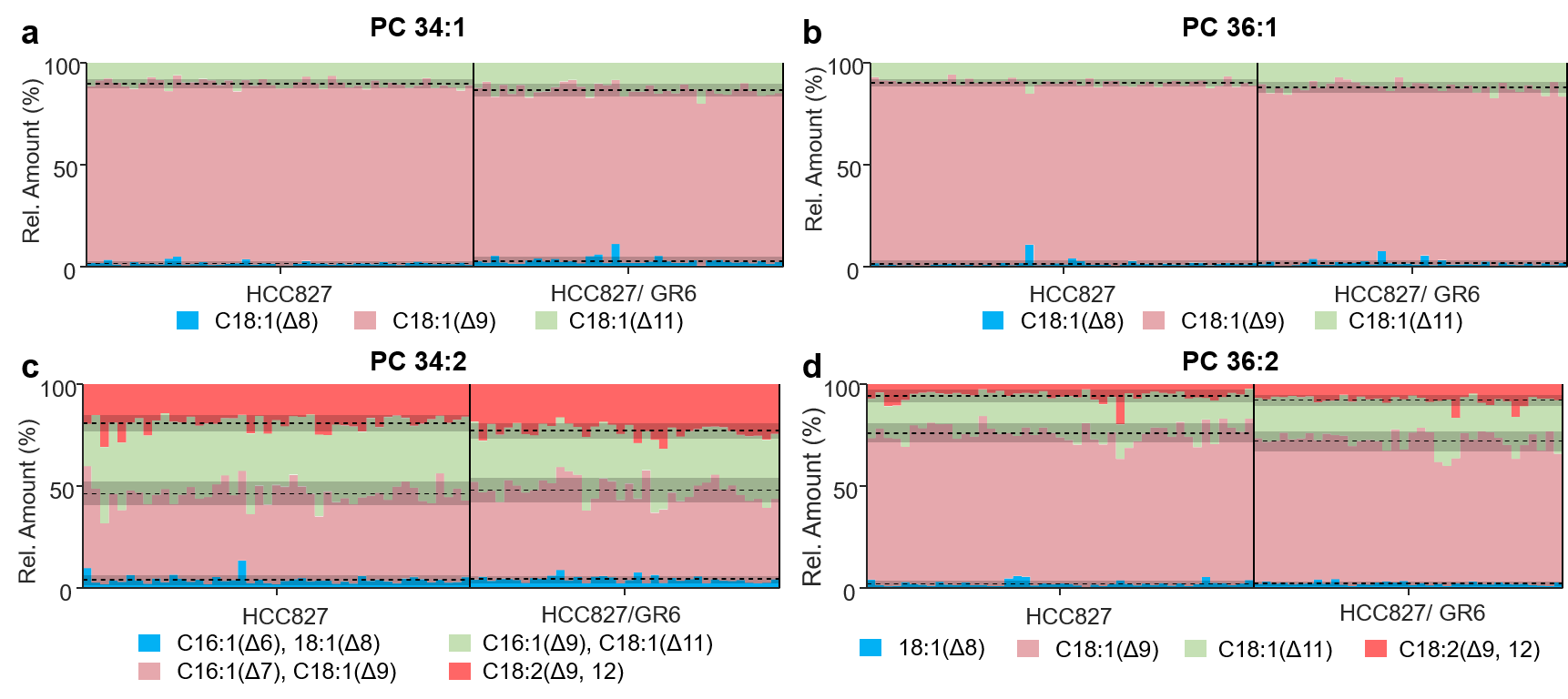


**Supplementary Fig. 24. Relative abundance of lipid C=C location isomers in HCC827 and HCC827/GR6 single cells. (a)** PC 34:1, **(b)** PC 36:1, **(c)** PC 34:2, **(d)** PC 36:2. HCC827: n = 45 single cells, HCC827/GR6: n = 36 single cells. Source data are provided in a Source Data file.

**Supplementary Table 16.** The significant difference for the relative amount of PC species with C=C locations between HCC827 and HCC827/GR6 single cells. The *p* values (calculated using two-tailed Student’s *t* test) highlighted in red indicate no statistical differences (*p*>0.05). HCC827: n = 45 single cells, HCC827/GR6: n = 36 single cells.

| Lipid species | HCC827 v.s. HCC827/GR6 |
| --- | --- |
| PC 16:0_16:1(Δ6) | 1.42E-08 |
| PC 16:0_16:1(Δ7) | 2.49E-13 |
| PC 16:0_16:1(Δ9) | 2.08E-16 |
| PC 16:1(Δ6)_18:1(Δ8) | 0.343110152 |
| PC 16:1(Δ7)_18:1(Δ9) | 0.260289053 |
| PC 16:1(Δ9)_18:1(Δ11) | 1.01E-07 |
| PC 16:0_18:2(Δ9, 12) | 2.15E-05 |
| PC 16:0_18:1(Δ8) | 0.000199342 |
| PC 16:0_18:1(Δ9) | 9.32E-11 |
| PC 16:0_18:1(Δ11) | 4.40E-07 |
| PC 18:1(Δ8)_18:1(Δ8) | 0.652247219 |
| PC 18:1(Δ9)_18:1(Δ9) | 0.00026629 |
| PC 18:1(Δ11)_18:1(Δ11) | 0.028704437 |
| PC 18:0_18:2(Δ9, 12) | 0.001283273 |
| PC 18:0_18:1(Δ8) | 0.496601807 |
| PC 18:0_18:1(Δ9) | 0.00014523 |
| PC 18:0_18:1(Δ11) | 1.32E-05 |


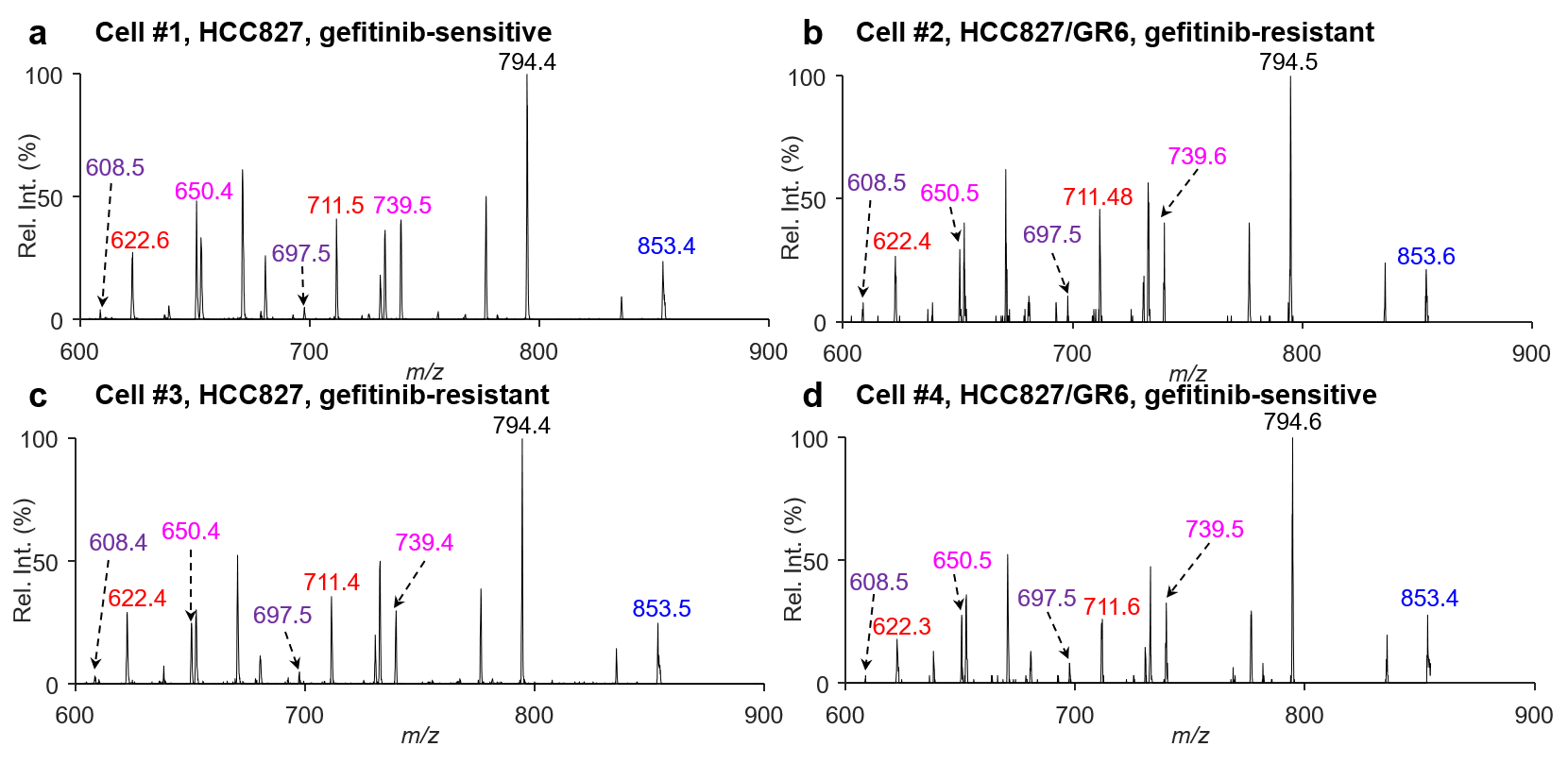


**Supplementary Fig. 25. Typical MS/MS spectrum of ^PB^(PC 32:1) in the marked four single cells in Fig. 6c and f. (a)** Cell #1, from HCC827 cell line and clustered to be gefitinib-sensitive; **(b)** Cell #2, from HCC827/GR6 cell line and clustered to be gefitinib-resistant; **(c)** Cell #3, from HCC827 cell line and clustered to be gefitinib-resistant; **(d)** Cell #4, from HCC827/GR6 cell line and clustered to be gefitinib-sensitive.

**Supplementary References**

1. Ntambi JM, Miyazaki M. Regulation of stearoyl-CoA desaturases and role in metabolism. *Prog Lipid Res* **43**, 91-104 (2004).

2. Kamphorst JJ*, et al.* Hypoxic and Ras-transformed cells support growth by scavenging unsaturated fatty acids from lysophospholipids. *Proc Natl Acad Sci USA* **110**, 8882-8887 (2013).

3. Vriens K*, et al.* Evidence for an alternative fatty acid desaturation pathway increasing cancer plasticity. *Nature* **566**, 403-406 (2019).
