# Supplementary material for: Single-cell lipidomics with high structural specificity by mass spectrometry
Source: Nat Commun. 2021 May 17;12:2869. doi: 10.1038/s41467-021-23161-5 (PMC8129106; doi:10.1038/s41467-021-23161-5)
Supplement: Supplementary file 2 — Reporting summary [file 41467_2021_23161_MOESM2_ESM.pdf]

nature research

Corresponding author(s): Xiaoxiao Ma, Zheng Ouyang

Last updated by author(s): Apr 14, 2021

Reporting Summary

Nature Research wishes to improve the reproducibility of the work that we publish. This form provides structure for consistency and transparency in reporting. For further information on Nature Research policies, see our Editorial Policies and the Editorial Policy Checklist.

Statistics

For all statistical analyses, confirm that the following items are present in the figure legend, table legend, main text, or Methods section.

n/a	Confirmed	
☐	☒	The exact sample size (n) for each experimental group/condition, given as a discrete number and unit of measurement
☐	☒	A statement on whether measurements were taken from distinct samples or whether the same sample was measured repeatedly
☐	☒	The statistical test(s) used AND whether they are one- or two-sided<br>*Only common tests should be described solely by name; describe more complex techniques in the Methods section.*
☒	☐	A description of all covariates tested
☒	☐	A description of any assumptions or corrections, such as tests of normality and adjustment for multiple comparisons
☐	☒	A full description of the statistical parameters including central tendency (e.g. means) or other basic estimates (e.g. regression coefficient) AND variation (e.g. standard deviation) or associated estimates of uncertainty (e.g. confidence intervals)
☐	☒	For null hypothesis testing, the test statistic (e.g. F, t, r) with confidence intervals, effect sizes, degrees of freedom and P value noted<br>*Give P values as exact values whenever suitable.*
☒	☐	For Bayesian analysis, information on the choice of priors and Markov chain Monte Carlo settings
☐	☒	For hierarchical and complex designs, identification of the appropriate level for tests and full reporting of outcomes
☒	☐	Estimates of effect sizes (e.g. Cohen's d, Pearson's r), indicating how they were calculated

Our web collection on statistics for biologists contains articles on many of the points above.

Software and code

Policy information about availability of computer code

Data collection	Analyst 1.6.3 from Sciex
Data analysis	Analyst 1.6.3 from Sciex, Matlab 2019b

For manuscripts utilizing custom algorithms or software that are central to the research but not yet described in published literature, software must be made available to editors and reviewers. We strongly encourage code deposition in a community repository (e.g. GitHub). See the Nature Research guidelines for submitting code & software for further information.

Data

Policy information about availability of data

All manuscripts must include a data availability statement. This statement should provide the following information, where applicable:

- Accession codes, unique identifiers, or web links for publicly available datasets
- A list of figures that have associated raw data
- A description of any restrictions on data availability

A reporting summary for this Article is available as a Supplementary Information file.
All data supporting the findings of this study are available from the corresponding authors upon reasonable request.
The source data for Figs. 1d, 2e, 4, 5 and Supplementary Fig. 2, 16-24 are provided in a Source Data file.
The raw data of MS spectrum in this article are available from Figshare (https://doi.org/10.6084/m9.figshare.14381258.v3).

Field-specific reporting

Please select the one below that is the best fit for your research. If you are not sure, read the appropriate sections before making your selection.

- [x] Life sciences
- [ ] Behavioural & social sciences
- [ ] Ecological, evolutionary & environmental sciences

For a reference copy of the document with all sections, see nature.com/documents/nr-reporting-summary-flat.pdf

Life sciences study design

All studies must disclose on these points even when the disclosure is negative.

Sample size	We used 43 MDA-MB-468 cells, 40 MCF-7 cells, 39 MDA-MB-231 cells, 41 BT-474 cells , 33 MDA-MB-468 cells dealt with CAY10566, 45 HCC827 cells and 36 HCC827/GR6 cells for lipid C=C location isomers quantitation, 42 MDA-MB-468 cells, 36 MCF-7 cells, 36 MDA-MB-231 cells, 38 BT-474 cells, 27 HCC827 cells and 35 HCC827/GR6 cells for lipid sn-position isomers quantitation, 25 HCC827 cells and 26 HCC827/GR6 cells for lipid sum composition quantitation. These samples were just used to demonstrate the possible application of the developed single-cell lipidomic approach, but not for discovery of the actual biomarkers. No sample size calculation was performed and the sample size of 25~42 showed a relative reproducibility of single-cell lipid analysis results.
Data exclusions	No data were excluded.
Replication	For PCs remained ratio quantitation experiment, 3 independent experiments were performed with identical results.<br>For optimizing experiment of acetylpyridine isomers, 3 independent experiments were performed with identical results.<br>For multiple round sampling experiments of a single cell, 5 independent experiments were performed with identical results.<br>For single cell isomers relative quantitation, tens of single cells were analyzed from 2 independent cell batches of each cell line and their results were consist. The conclusion we made was based on all single-cell data acquired from different batches.
Randomization	For breast cancers, cells were grouped to 4 kinds of cell lines. For SCD1 inhibition experiment, cells were grouped into MAD-MB-468/CAY10566 groups according to whether the drug was used. For lung cancer cells, cells were grouped to HCC827 and HCC827/GR6 groups according to the cell line and to gefitinib-resistant and gefitinib-sensitive groups according to the t-SNE cluser results. No further grouping was applied.
Blinding	Blinding was not applied, because the demonstrations in different cell lines were just to show the differences among these cell lines.

Reporting for specific materials, systems and methods

We require information from authors about some types of materials, experimental systems and methods used in many studies. Here, indicate whether each material, system or method listed is relevant to your study. If you are not sure if a list item applies to your research, read the appropriate section before selecting a response.

Materials & experimental systems

n/a	Involved in the study	
x		Antibodies
	x	Eukaryotic cell lines
x		Palaeontology and archaeology
x		Animals and other organisms
x		Human research participants
x		Clinical data
x		Dual use research of concern

Methods

n/a	Involved in the study	
x		ChIP-seq
x		Flow cytometry
x		MRI-based neuroimaging

Eukaryotic cell lines

Policy information about cell lines

Cell line source(s)	MCF-7 cells were purchased from National Infrastructure of Cell Line Resource (Beijing, China). BT-474, MDA-MB-468 and MDA-MB-231 were obtained from Shanghai Enzyme Research Biotechnology Co. Ltd. (Shanghai, China). HCC827 and HCC827/GR6 cells used in this study were kindly gifted by Dr. Pasi A. Jänne (Dana-Farber Cancer Institute, Boston, MA). The HCC827 cells were originally obtained from American Type Culture Collection (ATCC; Manassas, VA).
Authentication	Cell lines authenticated by short tandem repeat (STR) testing.
Mycoplasma contamination	The cell lines were not tested for mycoplasma contamination.
Commonly misidentified lines (See ICLAC register)	No commonly misidentified lines used.
